# Engineering Mesoporous Silica Hosts for Ultrasmall ZnO Nanoparticles: A Dendritic Polymer-Assisted Strategy Towards Sustainable, Safe, and Effective Antibacterial Systems

**DOI:** 10.3390/nano15221697

**Published:** 2025-11-09

**Authors:** Aggeliki Papavasiliou, Kyriaki Marina Lyra, Elias Sakellis, Albany Milena Lozano Násner, Jose Gallego, Fotios K. Katsaros, Zili Sideratou

**Affiliations:** 1 Institute of Nanoscience and Nanotechnology, National Center for Scientific Research ‘‘Demokritos”, 15310 Aghia Paraskevi, Greece; k.lyra@inn.demokritos.gr (K.M.L.); e_sakel@phys.uoa.gr (E.S.); f.katsaros@inn.demokritos.gr (F.K.K.); 2 Condensed Matter Physics Section, Physics Department, National and Kapodistrian University of Athens, Panepistimiopolis, Zografos, 15784 Athens, Greece; 3 Association for Research and Development of Innovations and Technologies for the Protection of Environmental, Social, and Cultural Heritage, 48 Avenue Mont-Rabeau, 06200 Nice, France; milena.nasner@arditec.net (A.M.L.N.); jose.gallego@arditec.net (J.G.)

**Keywords:** ZnO nanoparticles, nanocomposites, mesoporous silica, hyperbranched polyethyleneimine, carboxy-methylated hyperbranched polyethyleneimine, hyperbranched polymers, dendritic polymer-assisted impregnation technique, antibacterial properties

## Abstract

In response to the urgent need for sustainable antibacterial solutions against antibiotic-resistant pathogens, this study presents a facile dendritic polymer-assisted approach for synthesizing highly active ZnO/mesoporous silica nanocomposites (SBA-15, SBA-16, KIT-6, MSU-X). Two hyperbranched polymers—polyethyleneimine (PEI) and carboxy-methylated polyethyleneimine (Trilon-P, TrP)—were employed as templating and metal-trapping agents. The influence of pore geometry, polymer functionality, and polymer-loading method (wet or dry impregnation) on ZnO nanoparticle (NP) formation was systematically examined. All nanocomposites exhibited high structural homogeneity, incorporating ultrasmall or amorphous ZnO NPs (1–10 nm) even at 8 wt.% Zn loading. Zn uptake was strongly dependent on polymer end groups, while the spatial distribution of ZnO NPs was dictated by the silica host structure. Antibacterial assays against *Staphylococcus aureus* revealed remarkable activity, particularly for ZnO/SBA-15_PEI, ZnO/SBA-16_PEI, and ZnO/MSU-X_TrP nanocomposites, with minimum inhibitory concentrations of 1–2.5 μg mL^−1^ Zn and over 90% mammalian cell viability. Life Cycle Assessment identified energy use as the main environmental factor, with ZnO/SBA-15_PEI_WI displaying the lowest impact. Overall, the interplay between silica pore architecture, polymer type, and impregnation method governs ZnO accessibility and bioactivity, establishing a versatile strategy for designing next-generation ZnO/SiO_2_ nanocomposites with tunable antibacterial efficacy and minimal cytotoxic and environmental footprint.

## 1. Introduction

Infectious diseases remain a major global threat, impacting both public health and economic stability. They account for over 20% of worldwide mortality, with bacterial infections alone being responsible for approximately 7.7 million deaths annually, underscoring the urgent need for sustainable and effective antibacterial strategies [1]. Compounding this challenge is the growing crisis of antibacterial resistance [2,3]. Antibiotics, which constitute the most common practice to treat these infections, present nowadays a limited efficacy due to the growth of antibiotic-resistant bacteria [4,5,6]. The improper and excessive prescription and use of antibiotics, coupled with the genetic flexibility of the bacteria, are identified as the primary cause for the appearance and prevalence of antibiotic resistance [4,5]. To tackle the hazards related to the escalation of infections and of antibiotic-resistant pathogens, inorganic nanoparticles have emerged as an efficient alternative solution to conventional antibiotics [4,6,7,8]. Au, Ag, CuO, TiO_2_, and ZnO NPs are amongst the most widely studied inorganic antimicrobial agents [7,8], with ZnO singled out as a highly potent material in view of its low cost, abundance, and long-term effectiveness [4,9,10,11]. Although ZnO nanoparticles (NPs) may still pose health risks depending on their concentration and route of exposure, such as skin contact, ingestion, or inhalation, ZnO in its bulk form is classified as ‘generally recognized as safe’ and is approved by the U.S. Food and Drug Administration (FDA) [12]. Despite their broad potential in diverse sectors, such as pharmaceuticals, cosmetics, textiles, and agriculture, the widespread use of ZnO NPs has been restricted due to concerns about their toxicological impact on both human health [13,14] and the environment [15,16].

The antibacterial effect of ZnO, bactericidal or bacteriostatic, relies on multiple mechanisms mediated by chemical and physical interactions with the bacterial cell [10]. Chemical interactions include the generation of reactive oxygen species (ROS) and the release of Zn^2+^ ions, while physical interactions mostly include electrostatic, hydrophobic interactions and van der Waals forces, leading to the attachment of ZnO nanoparticles onto the bacterial cell wall [10]. The antimicrobial mechanisms can act simultaneously and induce cell envelope rupturing, cellular internalization, oxidative stress, and interaction and impairment of intracellular components, such as DNA, lysosomes, ribosomes, enzymes, etc. [7,8,9,10,11,17].

Properties of ZnO nanoparticles, such as shape/morphology, size, and surface modification, are considered key factors determining, to a great extent, their antimicrobial effect [10,17,18,19,20]. Downsizing ZnO particles to the nanoscale region has proved a highly beneficial approach in view of the size-dependency of several ZnO properties [8,10,17]. Particularly, oxygen defect-induced ROS production, dissolution of ZnO NPs into Zn^2+^, accumulation on the bacterial cell membrane, and ZnO internalization, all contributing towards antibacterial activity, are notably enhanced by decreasing particle size [10,17,21]. A clear correlation between ZnO particle size, cellular internalization and ROS generation was reported by G. Applerot et al. [21]. According to their findings, nanoscale ZnO particles displayed pronounced antibacterial activity, attributable to their facile penetration into bacterial cells and the subsequent formation of ROS, predominantly OH radicals, resulting in bacterial cell damage. Notably, this antibacterial mechanism was not observed with microscale ZnO particles.

Yet, despite their promising features, ZnO NPs present some significant drawbacks associated with cytotoxicity and ecotoxicity effects in a dose-dependent manner, as previously mentioned, and with the great aggregation tendency, induced either by their intrinsic nature or by the defense bacteria mechanism [11,19,22,23,24]. Lately, the encapsulation or immobilization of inorganic nanoparticles on mesoporous silica hosts is adopted as an effective strategy to address these issues [25,26,27]. Ordered mesoporous silicas, owing to their appealing textural features, such as high surface area, large pore volume, well-defined porous network, and adjustable pore size in the nanometer range—coupled with their biocompatibility, high loading capacity, and facile functionalization due to the silanol-enriched surface—have been established as ideal nanocarriers for drug delivery and currently for antibacterial agents [28,29,30,31,32]. Still, in order to ensure a high NP dispersion state, apart from the utilization of porous media, special attention must be given to the deposition procedure employed. Up to now, significant advances have been made towards engineering mesoporous silicas with metal or metal oxide NPs for a wide variety of applications. To this end, numerous methods have been applied, including wet impregnation, solid-state mixing, precipitation, co-condensation, template ion-exchange, and post-grafting [33]. Conventional impregnation or precipitation techniques, although quite simple to conduct, usually lack control over the final NP size and dispersion [33]. Direct incorporation of metal ions via co-condensation routes affords uniform metal distribution; however, it also results in insufficient material use, since a large amount of metal is buried within the siliceous skeleton. On the other hand, silica functionalization with organic molecules able to entrap metal ions can yield easily accessible and finely dispersed metal species [33]. However, only limited work has been reported referring to the antibacterial properties of such systems. Specifically, Vallet-Regí and co-workers in their work describe a synthetic strategy for the design of a multicomponent antimicrobial nanosystem, combining the effect of an antibiotic molecule and of antimicrobial Zn^2+^ or Ag^+^ ions [34]. In this case, a third-generation polypropyleneimine dendrimer was employed for the external silica functionalization, enabling not only an efficient internalization in Gram-negative bacteria but also an effective decoration of the silica surface with Zn^2+^ and Ag^+^ ions through complexation with the tertiary amine groups. In another study, Kankala et al. developed an antibacterial nanocomposite by externally depositing a hyperbranched polyethyleneimine/Ag NPs complex layer on copper-doped mesoporous silica already loaded with tetracycline guest molecules [35]. Moreover, the group of Zhou has focused on the production of antibacterial nanomaterials through the modification of mesoporous silicas with specific organic moieties forming coordinate bonds with several metal ions, including Zn^2+^ and Cu^2+^, affording at the end ZnO and/or CuO NPs either deposited or embedded into the siliceous matrix [36,37].

In this context, a novel facile dendritic polymer-templating strategy is proposed for the fabrication of ZnO NPs/mesoporous silica antibacterial nanocomposites. Hyperbranched polymers possess a key role in this strategy, serving as metal-trapping and -templating agents and hence enabling the geometric restriction of ZnO NPs within the siliceous mesoporous frameworks in an easy and precise manner. Specifically, by introducing these polymers into mesoporous silicas, abundant fixed-metal-anchoring sites, through metal–ligand complex formation, are furnished, controlling in this way the size, dispersion, and location of the generated ZnO NPs. In the past, our group has produced a number of novel nanoporous catalytic materials by harnessing hyperbranched polymers’ intriguing chemical and chelating properties [33,38,39,40]. Herein, this knowledge is transferred to the formulation of antibacterial nanocomposites and further enriched by investigating the effect of the siliceous hosts’ pore geometries in combination with the method of polymer’s loading and the type of its terminal functional groups, on the final ZnO NPs properties. For this purpose, four mesoporous silicas with different pore structural characteristics were surveyed as hosts, i.e., SBA-15, SBA-16, KIT-6, and MSU-X. SBA-15 possesses large, uniform mesopores arranged in a two-dimensional hexagonal symmetry with intra-wall micropores [41]. SBA-16 exhibits a three-dimensional body-centered cubic structure (Im3m) with interconnected cage-like mesopores, enhancing molecular accessibility and mass transport compared to the unidirectional channels of SBA-15 [42]. MSU-X displays a less ordered wormhole-like framework with uniform channels and thick pore walls, enhancing reactants diffusion rate and enabling synthesis under near-neutral pH conditions [43]. KIT-6 exhibits a cubic Ia3d symmetry with interpenetrating bicontinuous channels, providing a highly open structure that facilitates transport of guest species without pore blockage [44]. In addition, two hyperbranched polymers were employed, specifically hyperbranched polyethyleneimine (PEI) and a carboxy-methylated hyperbranched polyethyleneimine (Trilon-P, TrP), which possess different functional end groups, i.e., primary amines and carboxylate groups, respectively. Polymers were introduced into the siliceous framework either through the wet- or dry-impregnation method. After thorough physicochemical characterization, the antibacterial activity of the resulting ZnO/SiO_2_ nanocomposites was evaluated against the Gram-positive bacterium *Staphylococcus aureus* (*S. aureus*), while their cytotoxicity was assessed in mammalian cell lines. In addition, Life Cycle Assessment (LCA) methodology was employed as a robust tool to examine the environmental impacts associated with the synthesis of the most effective ZnO/SiO_2_ nanocomposites, which demonstrated superior antibacterial performance.

## 2. Materials and Methods

### 2.1. Chemicals and Reagents

Hyperbranched polyethyleneimine (PEI) of 5000 Da molecular weight (Lupasol^®^ WF, water-free, 99%) and carboxy-methylated polyethyleneimine (Trilon-P, TrP) were kindly donated by BASF (Ludwigshafen, Germany). Sodium trisilicate solution (Water glass), tetraethyl orthosilicate (TEOS) 98%, pluronic^®^ F-127, pluronic^®^ P-123, TWEEN^®^ 60, zinc nitrate hexahydrate (≥99%), HCl (37%), glutaraldehyde (solution, 25%), sodium cacodylate sodium chloride (NaCl), sodium hydroxide (NaOH), sodium fluoride (NaF), tryptic soy broth (TSB), and agar were purchased from Sigma-Aldrich (St. Louis, MA, USA). RPMI 1640 medium was purchased from Biowest (Nuaillé, France). Dulbecco’s phosphate-buffered saline (PBS), penicillin/streptomycin, trypsin/EDTA, and fetal bovine serum (FBS) were obtained from Biochrom GmbH (Berlin, Germany). Thiazolyl blue tetrazolium bromide (MTT) and isopropanol were purchased from Merck KGaA (Calbiochem^®^, Darmstadt, Germany). Peptone from Casein was purchased from AppliChem GmbH (Darmstadt, Germany).

### 2.2. Synthesis of Mesoporous Silica Hosts

The synthetic protocols employed for the mesoporous silica hosts’ production are based on hydrothermal routes with the aid of commercially available non-ionic triblock poly(ethylene oxide)-poly(propylene oxide)-poly(ethylene oxide) (PEO-PPO-PEO) copolymers as structure-directing agents (Pluronic P123 and F127), in a strong acidic or mild acidic environment. Specifically, the synthetic protocol selected for SBA-15, described in the work of Kosuge et al. [41], utilizes low-cost water-soluble sodium silicate as the silica source without hydrothermal treatment of the obtained solid. Therefore, this route offers many advantages, such as lower costs, shorter time, and simplicity in preparation, especially when intended for commercial applications. In brief, P123 (1.739 g) was dissolved in 68 mL of 2 M HCl (mixture A), and a dilute sodium silicate solution was prepared, consisting of 4 g sodium silicate solution (≈26.5 wt.%) and 12 g of deionized water (DW, mixture B). Mixture A was added to mixture B, with the temperature maintained at 30 °C and stirring at 600 rpm applied only for 30 s. The reaction solution was subsequently kept under static conditions for 6 h. The obtained solid was filtrated and washed repeatedly with warm DW (~300 mL warmed at 30–35 °C). After drying at room temperature for 2 days and then at 50 °C for another 2 days, the product was calcined at 550 °C for 6 h at a heating rate of 1 °C min^−1^.

For the production of SBA-16, the synthetic procedure proposed by Grudzien et al. [42] was followed, involving the dissolution of Pluronic F127 (2 g) and sodium chloride (7.05 g) into a solution containing 20 mL of HCl (2 M) and 60 mL DW at 40 °C. Then, TEOS (8.4 g) was added dropwise and left stirring for 20 h at 40 °C. The produced sol was hydrothermally treated for 24 h at 100 °C under static conditions. The precipitate was filtered, washed with DW, dried in an oven at 80 °C, and then calcined under the same conditions as previously mentioned in the case of SBA-15.

KIT-6 was produced through the synthetic route reported by Kleitz et al. [44]. Typically, 1.94 g of P123 were dissolved in 70 mL of distilled water and 3.2 mL of HCl (37 wt.%). After complete dissolution, 2.4 mL of butanol was added. The mixture was left stirring at 35 °C for 1 h, followed by the addition of TEOS (4.16 g). After another 24 h of stirring at 35 °C, the mixture was hydrothermally treated at 100 °C for 24 h under static conditions. The solid product was filtered without washing, dried at 100 °C, and calcined under the same conditions as in the case of SBA-15.

A modified synthetic protocol based on the one described by Boissiere et al. [43] was adopted for the synthesis of MSU-X. First, an acidified aqueous solution of Tween 60 (0.02 M) was prepared with a proper amount of dilute HCl to adjust the pH to 2.3. Then, the solution was magnetically stirred at mild heating (45 °C) until homogenization, followed by the addition of TEOS (molar Si/surfactant = 8). After about 2 h to allow for hydrolysis, sodium fluoride (molar NaF/Si = 0.04) was introduced to this solution to initiate silica condensation and left stirring for 30 min. Subsequently, the obtained mixture was placed in a thermostated water bath for 24 h at 45 °C without any disturbance of the solution. Finally, the derived material was filtrated, dried, and calcined at 550 °C for 6 h at a heating rate of 1 °C min^−1^.

### 2.3. Synthesis of ZnO/SiO_2_ Nanocomposites

**Scheme 1:**
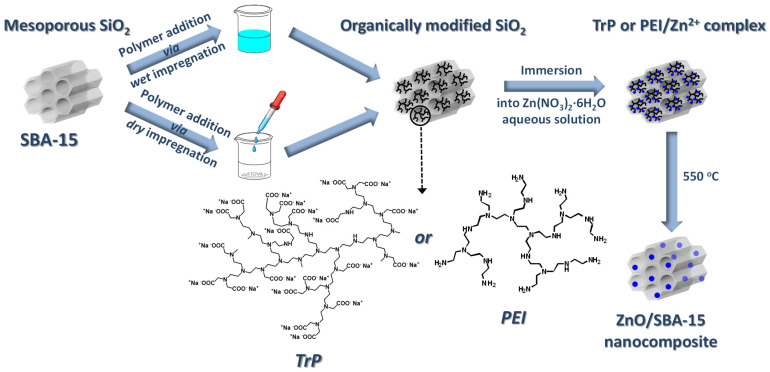
Schematic illustration of ZnO/SiO_2_ nanocomposites synthesis via a dendritic polymer-templating route.

For the production of the ZnO NPs/mesoporous silica nanocomposites, a dendritic polymer-templating route was employed, which comprises the following synthetic steps, as illustrated in Scheme 1.

At first, organic modification of the different siliceous supports took place with the introduction of the hyperbranched polymers using wet or dry impregnation procedures. This modification is based on the polymers’ physical attachment onto the silica surface via electrostatic interactions and/or hydrogen bonding and thus requires only a 24 h outgassing of the powder as a pretreatment step so as to facilitate the polymer’s penetration into the porous network. In the case of wet impregnation, 500 mg of silica powder was suspended in a polymeric aqueous solution (0.2 wt.%) and left stirring at room temperature (RT) for 24 h. In contrast, in the case of dry impregnation, a polymer solution of appropriate volume (depending on the pore volume of each silica) was added dropwise to the silica powder with simultaneous stirring until a homogeneous paste was formed. In both cases, given the high aqueous solubility of the hyperbranched polymers employed herein, the obtained polymer/silica materials were washed with DW to take off the free hyperbranched macromolecules accumulated mainly at the external SBA-15 surface.

For metal sorption, 350 mg of the dried organically modified mesoporous silicas were dispersed in 100 mL of zinc nitrate hexahydrate aqueous solution (Zn(NO_3_)_2_·6H_2_O) with an initial concentration (C_0_) of 10 g/L and then stirred for 24 h at RT. Solution’s pH was adjusted around 5 to 5.5 in view of reaching the highest Zn adsorption capacity avoiding at the same time the precipitation of Zn in the form of hydroxides, Zn(OH)_2_. More precisely, at low pH values hyperbranched polymer’s protonation impedes metal binding, whereas at pH values approaching the precipitation limit, the formation of zinc oxo-species becomes unavoidable. Therefore, maintaining this specific pH range ensures a controlled ZnO growth mechanism mediated solely by the hyperbranched polymer. Following the 24 h sorption, solids were filtered, washed, dried, and calcined at 550 °C for 5 h with a heating rate of 1 °C min^−1^ under air flow for the decomposition of the hyperbranched polymers and the acquisition of the final ZnO-loaded silicas.

### 2.4. Materials Characterization

Thermogravimetric analysis was carried out on a Setaram SETSYS Evolution 18 thermal analyzer (Setaram Instrumentation, Caluire, France), with a heating rate of 10 °C min^−1^, in an alumina crucible and by employing dried air as the carrier gas. FTIR spectroscopy was performed over the range of 4000–500 cm^−1^ using a Thermo Scientific Nicolet 6700 FTIR (Thermo Scientific, Waltham, MA, USA), equipped with a Specac Quest ATR (Attenuated Total Reflection) having a diamond crystal (Specac Ltd., Orpington, Kent, UK). For each sample, 128 scans were averaged at a resolution of 4 cm^−1^, with all spectra being normalized against a single-beam spectrum of the clean ATR crystal and converted into absorbance units. Low- and wide-angle XRD diffraction patterns were acquired using a Rigaku rotating anode X-ray generator (operating at 50 kV, 100 mA, using Ni-filtered Cu Ka1 radiation) equipped with an R-AXIS IV image plate (Rigaku Co., Tokyo, Japan). All samples were mounted in Lindemann capillaries (Hilgenberg-Mark tubes of 0.7 mm inner diameter). N_2_ adsorption measurements were performed at 77 K on an automated volumetric system (AUTOSORB-1-Krypton version—Quantachrome Instruments, Boynton Beach, FL, USA). Prior to measurements, samples were degassed at 250 °C for 12 h. The specific surface area was determined using the Brunauer–Emmet–Teller method. The pore size distribution was calculated using the Nonlocal Density Functional Theory (NLDFT) model (Quantachrome Instruments, AS1Win software, Version 2.01, 2016). Data reduction parameters: Calc. Model: N_2_ at 77 K on silica (cylindrical pore, NLDFT equilibrium model or adsorption branch). SEM analysis was conducted on a JEOL JSM 7401F Field Emission Scanning Electron Microscope (JEOL, Tokyo, Japan) equipped with a Gentle Beam mode operating at 2 kV acceleration voltage. The samples were placed on metallic (brass) substrates with double-coated carbon conductive tape. For the Si, Zn, and O elemental mapping analysis and determination of the final Zn loading, an EDS microanalysis was employed at a 20 kV acceleration voltage, utilizing an Xplore-15 SDD detector (Oxford Instruments, Abingdon, UK) with a 15 mm^2^ surface area. TEM, HRTEM, STEM imaging, along with Si, Zn, and O elemental mapping distributions at the nanoscale, were obtained via a FEI Talos F200i field-emission (scanning) transmission electron microscope (Thermo Fisher Scientific Inc., Waltham, MA, USA), operating at 200 kV. The microscope was equipped with a windowless energy-dispersive spectroscopy microanalyzer (6T/100 Bruker, Hamburg, Germany). TEM samples were prepared by dispersing the sample in ethanol and then drop-casting it onto a carbon-coated Cu grid.

### 2.5. Evaluation of Antibacterial Activity

The antibacterial activity of Zn-loaded nanoporous silica materials was studied against Gram (+) *Staphylococcus aureus* (*S. aureus* strain ATCC 25923) following the CLSI guidelines (documents M07-A9 and M26-A) [45,46]. Bacteria were grown at 37 °C in tryptic soy broth (TSB) for 16 h, in aerobic conditions, using a Stuart SI500 orbital shaker (~200 rpm shaking speed, Bibby Scientific Ltd., Staffordshire, UK). Then, the bacterial suspension was diluted with TSB to a concentration equal to 0.5 McFarland Standard (~10^8^ CFU/mL) as confirmed by measuring their absorbance at 600 nm on a Cary 100 Conc UV–vis spectrophotometer (Varian Inc., Mulgrave, Australia). The resulting dispersion was one-fold-diluted and used for the subsequent tests.

*Minimum Inhibitory Concentration (MIC) determination*: The broth macro-dilution method was employed to calculate the MIC values for all ZnO-loaded mesoporous silicas, following the standard CLSI M07-A9 protocol [46]. Specifically, dispersions of ZnO-loaded mesoporous silicas in TSB were prepared at various concentrations ranging from 25 to 1700 µg/mL. Subsequently, 20 μL of bacterial suspension containing ~10^−7^ CFU/mL of *S. aureus* was inoculated into 2 mL of each dispersion of mesoporous silicas and incubated in a Stuart SI500 orbital shaker (~200 rpm rotation speed) at 37 °C for 16 h. Untreated bacteria and bacteria-free growth media served as positive and negative controls, respectively. Following the incubation period, the MIC was defined as the lowest concentration at which no visible bacterial growth was detected.

*Minimum Bactericidal Concentration (MBC) determination*: The colony-counting method was used to determine the MBC values for all ZnO-loaded mesoporous silicas, following the standard CLSI M26-A protocol [45]. In brief, 100 μL of aliquots were taken from the tube at the MIC value and from tubes at three higher concentrations than MIC, one-fold-diluted, and plated on agar plates. After incubation at 37 °C for 24 h, bacterial colonies (CFU/mL) were counted, and the reduction in bacterial growth was calculated for each sample. The MBC was defined as the lowest concentration that resulted in a 99.9% reduction in the initial bacterial inoculum. All experiments for MIC and MBC determination were conducted at least in triplicate to ensure reproducibility.

*Morphological analysis of bacteria*: To determine the morphology of *S. aureus* bacteria after treatment with ZnO-loaded mesoporous silicas, Scanning Electron Microscopy (Jeol JSM 7401F Field Emission SEM, Jeol, Tokyo, Japan) was used. Briefly, bacteria were incubated with mesoporous silica materials at a concentration equal to MIC. After overnight incubation, bacteria were fixed with 3% glutaraldehyde in sodium cacodylate buffer (100 mM, pH = 7.1) for 12 h. Subsequently, the bacteria were collected by centrifugation as a pellet, washed, and resuspended in sodium cacodylate buffer. Subsequently, a 50 μL aliquot of each suspension was placed on poly(L-lysine)-coated glass coverslips. Samples were dehydrated in graded ethanol (50%, 70%, 95%, 100%, 10 min each), air-dried, and coated with gold using a sputter coater prior to SEM imaging [47,48].

### 2.6. Evaluation of In Vitro Cytotoxicity

The cytotoxicity of ZnO-loaded mesoporous silicas was evaluated using the standard MTT assay. Human embryonic kidney cells (HEK293) and human prostate cancer cell lines (PC3) were cultured in an RPMI-1640 medium supplemented with 10% fetal bovine serum (FBS) and penicillin (100 U/mL)/streptomycin (100 μg/mL). Cells were maintained at 37 °C in a humidified atmosphere with 5% CO_2_ and sub-cultured twice weekly using a trypsin (0.05% *w*/*v*)/EDTA (0.02% *w*/*v*) solution.

For the MTT assay, 10^4^ cells/well were seeded in 96-well plates and incubated in complete medium for 24 h. Following this pre-incubation period, cells were treated with ZnO-loaded mesoporous silicas at MIC-related concentrations ranging from 50 to 500 µg/mL (total material) for 24 h (the same exposure time used in the antibacterial tests). Subsequently, the medium was replaced with 100 μL of MTT solution (10 μg/mL in complete RPMI) and incubated for an additional 4 h under the same conditions. Formazan crystals formed during this period were dissolved in 100 μL of 2-propanol per well, and absorbance was measured at 540 nm using an Infinite M200 microplate reader (Tecan Group Ltd., Männedorf, Switzerland).

Each concentration was tested in six replicates, and each experiment was repeated three times independently. Cell viability (%) was calculated relative to untreated controls (cells incubated in complete medium). Blank values from wells containing only 2-propanol were subtracted from all measurements.

To evaluate the statistical significance of differences in cell viability, a two-tailed paired Student’s *t*-test was performed comparing treated samples to controls. Statistical significance was categorized as follows: *p* < 0.05 (*), *p* < 0.01 (**), *p* < 0.001 (***), *p* < 0.0001 (****), and not significant (ns) for *p* > 0.05.

### 2.7. LCA of the ZnO/SiO_2_ Nanocomposites

Environmental impacts of ZnO/SiO_2_ nanocomposites with superior antibacterial performance were evaluated using standardized Life Cycle Assessment (LCA). The methodology encompassed defining the goal and scope, compiling the life cycle inventory (LCI), performing the life cycle impact assessment, and interpreting the results, quantified through SimaPro 9.3 software. The functional unit refers to the production of 1 g of nanocomposite (total mass without considering the antibacterial activity), and the inventory data belongs to the laboratory-scale formulation (Table 1). The impact assessment method used was the Environmental Footprint 3.1 Method (EF3.1 adapted), as recommended by the European Commission (Commission Recommendations 2013/179/EU) [49].

## 3. Results and Discussion

### 3.1. Characterization of the Pristine Mesoporous Silicas

The pure siliceous materials employed as hosts for ZnO NPs were characterized using a combination of techniques, including low- and wide-angle XRD, nitrogen adsorption–desorption isotherms, and scanning electron microscopy (SEM). As derived by the low-angle XRD analysis (Figure S1A), SBA-15, SBA-16, and KIT-6 samples demonstrate order mesoporous structures of very high quality. Specifically, the low-angle XRD pattern of SBA-15 material exhibits three diffractions corresponding to the (100), (110), and (200) planes of 2D hexagonal *p*6*mm* structure; in the case of SBA-16, the one major and the two less intensive peaks are indexed as (110), (200) and (211) reflections in the cubic space group (*Im* $\bar{3}$*m*), while in KIT-6 pattern all the characteristic reflections ascribed to the cubic *Ia* $\bar{3}$*d* symmetry could be identified [41,42,43]. In contrast, in the case of MSU-X, only a broad diffraction peak could be detected, indicative of the disordered worm-hole-like structure of MSU-X-type materials [44]. Wide-angle XRD diffractograms of all silica samples (Figure S1B) present only a single very broad low-intensity peak around 22°, denoting the amorphous nature of the pore walls.

Additional information about the pore architecture was obtained by N_2_ adsorption analysis. The derived N_2_ adsorption–desorption isotherms together with the corresponding pore size distribution curves are shown in Figure S2, whereas N_2_ sorption properties, such as specific surface area, total pore volume, and average pore size, are summarized in Table 2. All materials’ isotherm plots are of type IV, as defined by IUPAC classification, typical of mesoporous solids [50]. However, differences, particularly in the hysteresis loops, can be distinguished. N_2_ sorption isotherms of SBA-15 and KIT-6 samples present a steep capillary condensation step and a H1 type hysteresis loop, revealing uniform mesochannels of cylindrical geometry (Figure S2A) [41,43]. This pore uniformity is further substantiated by the pore size distribution (PSD) analysis, calculated by applying the nonlocal density functional theory (NLDFT) equilibrium method on the desorption branch. As seen in Figure S2B, monomodal, narrow, and symmetrical PSD curves are obtained, located though in different pore diameters, larger for KIT-6 material at about 8.5 nm. In the case of the SBA-16 sample, a broad H2 type hysteresis loop is observed with a delayed and steep desorption at ≈0.42 P/P_0_, related to ink-bottle or cage-like pore geometry (Figure S2A). This type of mesoporous material consists of cage-like mesopores interconnected by rather narrow pore entrances [42]. Pore size distribution (PSD) is estimated by applyingthe NLDFT method, using the model isotherm of N_2_ adsorbed on silica with cylindrical pores acquired from the adsorption branch (Figure S2B). According to the obtained PSD, the SBA-16 material consists of uniform mesopore bodies/cages with a mean size around 6.5 nm and narrow pore apertures smaller than 2.5 nm. The MSU-X sample demonstrates a smaller, ill-defined hysteresis loop (Figure S2A), located at lower P/P_0_ values, indicative of less uniform and smaller mesopores, also confirmed via the PSD analysis calculated by applying the NLDFT equilibrium method on the desorption branch (Figure S2B) [50]. As a result of the smaller pores, this sample possesses the largest specific surface area (SSA) value (1001 m^2^/g), while SBA-15, demonstrating the lower microporosity content, possesses the lowest specific surface area (SSA) value. Regarding the total pore volume (TPV) values, the KIT-6 sample with the largest pores presents the highest TPV value (1.16 cc/g).

**Figure 1:**
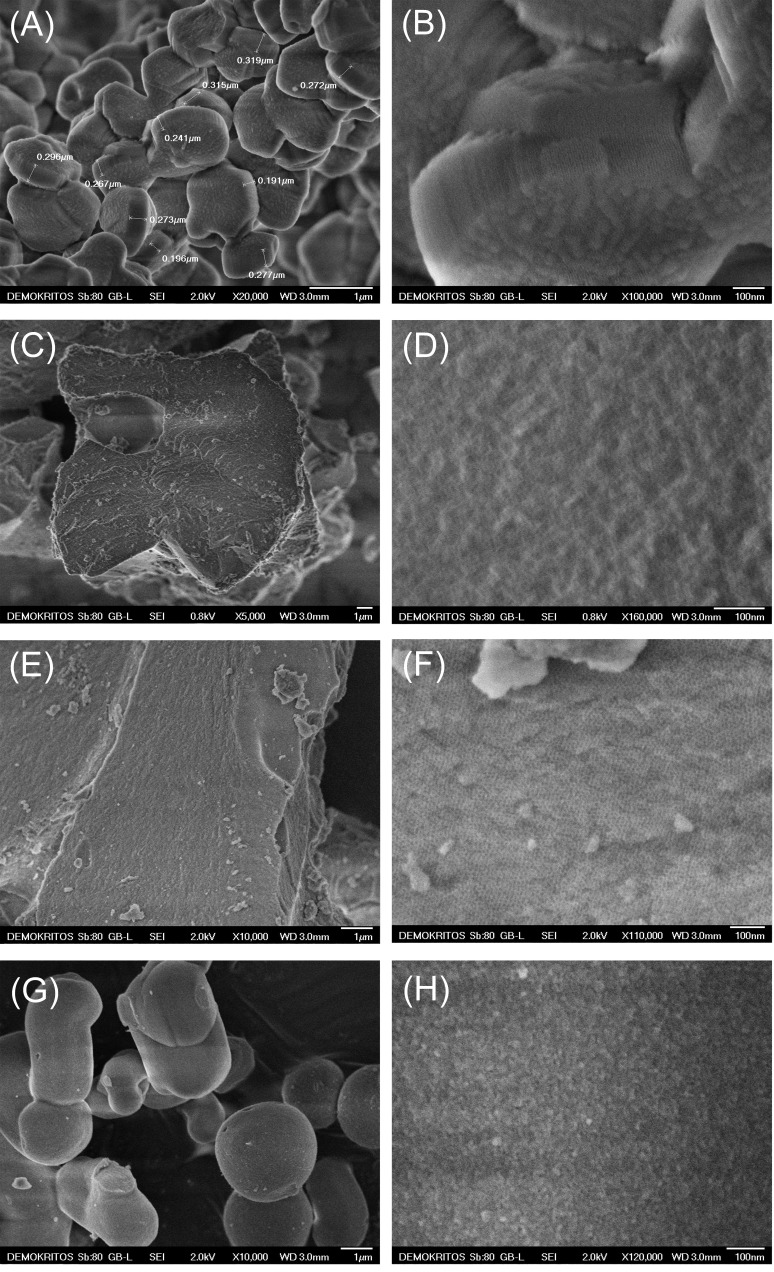
SEM micrographs of SBA-15 (**A**,**B**), KIT-6 (**C**,**D**), SBA-16 (**E**,**F**), and MSU-X (**G**,**H**) mesoporous hosts.

Morphological properties were investigated by means of scanning electron microscopy. SEM micrographs of the SBA-15 sample, depicted in Figure 1A,B, reveal a highly homogeneous morphology of loosely aggregated plate-like particles. At higher magnification, (Figure 1B), well-ordered channels of very short length, not exceeding 300 nm, and aligned along the thickness of the platelets could be distinguished. KIT-6 and SBA-16 samples consist of dense monolithic pieces with orderly arrayed mesopores, as detected in SEM micrographs of higher magnification, in line with the XRD and N_2_ adsorption findings (Figure 1C–F). Finally, the MSU-X material presents a mixed morphology containing micrometric-sized particles of different shapes, including curved rods and spheres (Figure 1G,H).

### 3.2. Characterization of Organically Modified Mesoporous Silicas

Organic modification of the different mesoporous siliceous networks, performed either through wet or dry impregnation techniques, involves physical attachment of the hyperbranched polymers onto the silica surface [40]. Particularly, hyperbranched Polyethyleneimine (PEI) is a highly branched cationic polymer bearing a large number of amino groups (primary, secondary, and tertiary) [33]. Accordingly, in this case, two characteristic interactions are expected: electrostatic between the protonated amine groups and surface Si–O^–^ groups and hydrogen bonding between the numerous end primary amino groups with the surface silanol groups [40]. Considering that the protonation degree of the polymers’ amino groups is strongly dependent on the pH environment: when wet impregnation was employed, the solution’s pH was appropriately adjusted through the addition of hydrochloride. The aim was to identify the conditions under which the stronger interaction, and thus the highest polymer uptake, was achieved. In the case of dry impregnation, a polymer solution of volume equal to the pore volume of the support material was slowly and gradually added, allowing for its insertion into the porous structure via capillary forces. Following the polymer’s addition, a washing procedure was performed to remove the free polymer from the external silica surface, and the final polymer loading was determined through TGA analysis.

In Figure S3, the TGA profiles of the SBA-15 sample modified with PEI through wet impregnation at three different pH values (pH = 3, 5.5, and 10) are presented. According to the weight losses recorded in the temperature region 200–650 °C due to the hyperbranched polymer thermal decomposition, it can be deduced that the alkaline pH environment (pH = 10) affords the highest polymer loading into the SBA-15 host. Most likely, at this pH value, the susceptible-to-hydrolysis Si-O-Si surface bonds result in a high hydroxylation degree, strengthening the polymer’s attachment through hydrogen bonding. In addition, under these conditions, the partially protonated amino groups of PEI electrostatically interact with the negatively charged silica surface, Si–O^–^ [39]. In view of this finding, the incorporation of PEI into the different siliceous hosts through wet impregnation was carried out at pH 10. Regarding the effect of the mesoporous host, as derived by TGA analysis (Figure S4), the highest polymer uptake (27%) was attained for KIT-6 sample, on account of its open pore structure featuring the highest pore volume and the largest pore diameter, thus favoring the polymer’s introduction. Similar results were also derived when dry impregnation was applied (Figure S5), with the KIT-6 sample demonstrating a final polymer uptake of the order of 30%. Moreover, in this case, polymer loading was increased in all mesoporous silicas, except for SBA-16, possibly due to its pore network comprising small pore openings.

Useful information on the polymer’s location within the different siliceous materials was drawn from N_2_ sorption measurements. Based on the values listed in Table 3, after hyperbranched polymer addition, a drastic drop in specific surface area and total pore volume is induced, together with an increase in the average pore diameter, for all materials investigated. This modification of the pore structural properties points to the successful polymer loading into the porous networks. This conclusion is further supported by the acquired N_2_-physisorption isotherms and pore size distribution (PSD) curves. As observed in Figures S6A–S9A, after polymer loading via wet impregnation, all materials display type IV isotherm plots accompanied by well-defined hysteresis loops, confirming the preservation of the pristine mesostructure. Only in the case of the SBA-16_PEI_WI sample is a transformation of the hysteresis loop type from H2 to H1 noticed. In addition, in all cases, the significantly smaller N_2_ uptake at low P/P_0_ values is typical of microporosity decrease. This is further corroborated by the obtained PSD curves (Figures S6B–S9B), which clearly demonstrate the reduction in microporosity resulting from polymer loading. In the mesopore area, a noticeable shift in the main PSD curve, in relation to the parent mesoporous materials, is detected for KIT-6- and SBA-16-polymer-modified samples. However, this shift is recorded towards different directions: at lower pore diameters in the KIT-6_PEI_WI sample and at larger pore diameters in the SBA-16_PEI_WI sample. Typically, guest species introduced into a mesoporous host generate a decrease in the pore diameter. The opposite finding, observed in the case of SBA-16, could derive from the strong modification of the pristine siliceous pore structure mostly due to the conditions of the loading procedure than to the polymer’s introduction. Finally, in the case of the SBA-15_PEI_WI and MSU-X_PEI_WI samples, the negligible shift in the main PSD curve compared to the parent siliceous materials, coupled with its substantial drop, could be attributed to both pore loading and plugging phenomena.

When dry impregnation was applied, a more pronounced modification of pore structural properties was produced, in line with the higher polymer uptake recorded using the TGA analysis. This is evidenced by both the significantly lower pore structural properties, summarized in Table 3, and the respective isotherm plots and PSD curves (Figures S10–S13). As noticed, in SBA-15_PEI_DI and MSU-X_PEI_DI samples, the hysteresis loop almost completely disappeared, and in the KIT-6_PEI_DI sample, the hysteresis loop and PSD curve were reduced and shifted to lower pore diameters compared to its counterpart derived by wet impregnation. On the contrary, the SBA-16_PEI_DI sample demonstrates the most well-preserved mesostructure, as witnessed by the shape of the isotherm plot and PSD curve, as well as by the reduced SSA and TPV losses, which is in complete agreement with its low polymer content, as determined via the TGA analysis. Moreover, according to this sample PSD, the curve at lower pore diameters corresponding to the pore entrances is more affected by the polymer addition than the respective curve assigned to the pore bodies, suggesting the polymer’s partial insertion into the SBA-16 network.

The second hyperbranched polymer investigated was carboxy-methylated polyethyleneimine (Trilon-P, TrP). This polymer was selected on account of the large number of methyl-carboxylated functional end groups and tertiary amino groups, endowing a strong chelating action towards a large variety of metal ions [40]. Trilon-P introduction into the different mesoporous hosts was once again performed through both wet and dry impregnation, followed by a washing step to take away the excess polymers. However, as deduced from the TGA analysis (Figures S14–S16), the final polymer content was significantly lower compared to silica samples modified with PEI. This observation can be rationalized by considering the molecular weight and anionic character of Trilon-P, the latter favoring its attachment mainly through hydrogen bonding. Specifically, under highly basic conditions, such as those of the initial Trilon-P solution, repulsive forces are exerted between the dissociated carboxylate groups of the polymer and the negatively charged silica surface. In addition, the relatively high molecular weight of Trilon-P (Mw ≈ 50,000 Da) and, consequently, its larger size likely represent an additional barrier to penetration into the siliceous pore network. The optimum pH value for wet impregnation, corresponding to the highest polymer uptake, was determined to be 5.5 (Figure S16).

Consistent with the low Trilon-P content, pore structural properties of the polymer-loaded silicas were not significantly altered, as evidenced by the well-preserved shapes of both the N_2_ sorption isotherms and PSD curves (Figures S17–S22). Moreover, particularly for the samples obtained via wet impregnation, the textural characteristics, including specific surface area and total pore volume, exhibited the smallest decrease among all samples investigated, while the average pore diameter showed the least enlargement (Table 3). This effect results not only from the extent of polymer loading but also from its spatial distribution, suggesting a possible decoration of the external silica surface. In contrast, N_2_ adsorption data indicate a higher degree of polymer incorporation into the siliceous pore network when dry impregnation was employed.

### 3.3. Characterization of ZnO/SiO_2_ Nanocomposites

As already shown in our previous work, hyperbranched polymers can act in an analogous manner as dendrimers, their almost symmetrical counterparts, effectively templating and stabilizing a large variety of metal/metal oxide NPs by sequestering metal ions in their interior. The mechanism of metal entrapping lies in complex formation between metal ions and internal tertiary amines, together with carboxyl end-groups, in the case of Trilon-P or end primary amines in the case of PEI, in a square planar coordination [40,51]. Therefore, through the modification of the mesoporous silicas with the hyperbranched polymers, specific metal sorption sites are generated. Then, by immersing the organically modified materials into an aqueous solution of zinc precursor, Zn^2+^ ions are immobilized on these positions, evolving to ZnO NPs with well-controlled properties upon thermal treatment. Besides the formation of ZnO NPs, thermal treatment at 500 °C is also applied to remove the organic content from the final material. Complete removal of the hyperbranched polymer after calcination is verified using the TGA analysis, with no weight loss recorded above 550 °C (Figure S23).

The Zn loadings in the synthesized ZnO/SiO_2_ nanocomposites were determined by SEM–EDS analysis, performed in multiple regions of each sample, and the results are summarized in Table 4. As observed, there is a good correlation between metal and polymer loadings, with only the ZnO/SBA-16_PEI_WI sample deviating from this trend. Zn content varies approximately between 1 wt.% to 8 wt.%, with the siliceous pore network also having a net effect. More precisely, higher Zn loadings were attained in the case of PEI and KIT-6 or MSU-X used as porous hosts, in virtue of their larger pore volume. Particularly, KIT-6 mesoporous silica has proven to be the optimum host to produce high Zn-loaded nanocomposites on account of its pore geometry. The 3D open-pore configuration with large cylindrical channels of KIT-6 host possibly enables the largest polymer uptake, affording consequently abundant, easily accessible metal sorption sites.

**Figure 2:**
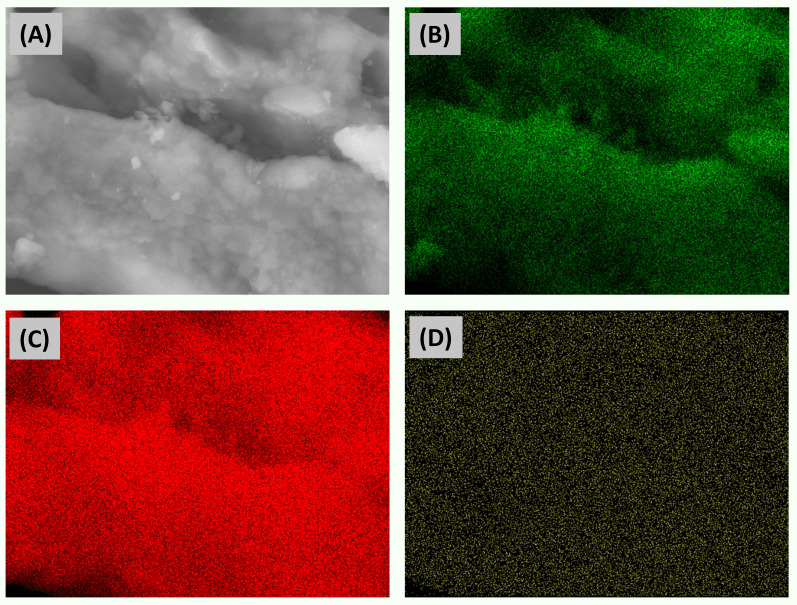
(**A**) SEM micrograph of ZnO/KIT-6_PEI_DI sample and the corresponding O (**B**), Si (**C**), and Zn (**D**) elemental maps.

SEM, along with elemental mapping measurements, was performed on all ZnO/SiO_2_ nanocomposites. As demonstrated by the obtained SEM micrographs, the Zn-loaded samples largely retained the pristine morphological features, i.e., dense monolithic pieces in the case of KIT-6 and SBA-16, loosely aggregated plate-like particles in the case of SBA-15 samples, and spherical-like in the case of MSU-X samples. Furthermore, EDS mapping confirmed an excellent spatial dispersion of Zn species across all samples, with no evidence of externally deposited ZnO aggregates. For brevity, the SEM–EDS results are presented only for the ZnO/KIT-6_PEI_DI sample, which exhibited the highest Zn loading (Figure 2), while the corresponding results for the remaining samples are provided in the Supporting Information (Figures S24–S36). Notably, only the ZnO/SBA-16_PEI_WI sample showed a change in morphology, with very small fragments decorating the silica surface which according to mapping analysis, could not be attributed to ZnO particles or aggregates.

**Figure 3:**
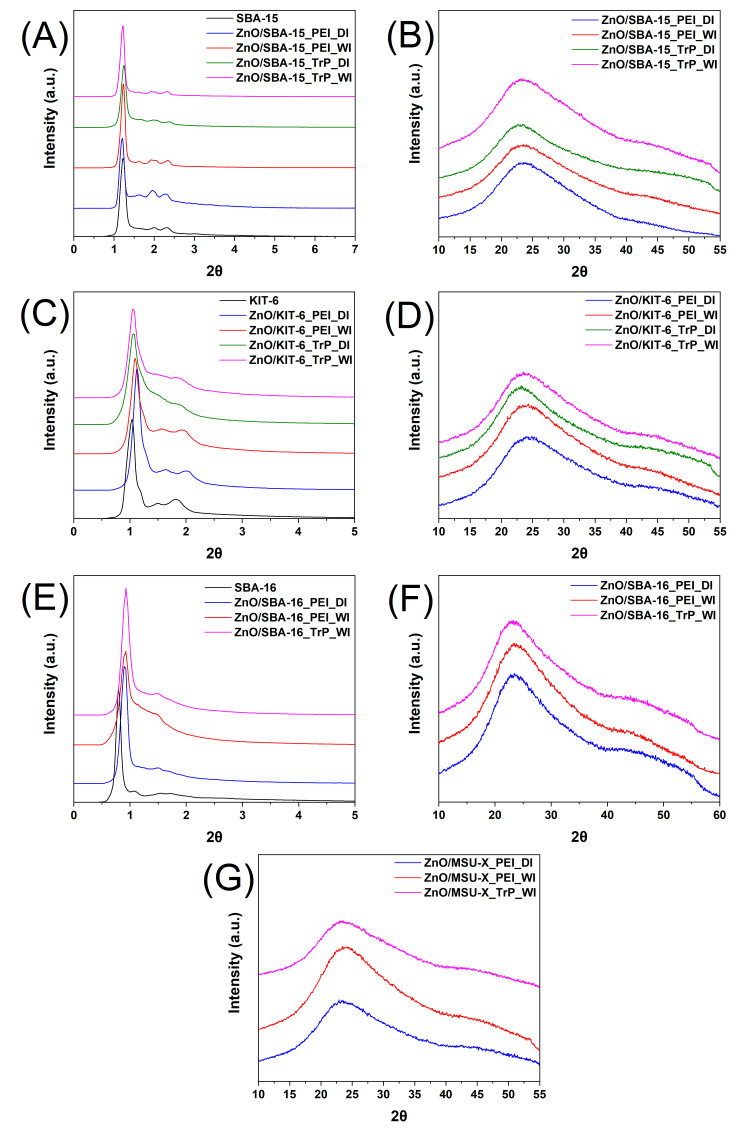
Low- (**A**,**C**,**E**) and wide-angle (**B**,**D**,**F**,**G**) XRD patterns of ZnO/SiO_2_ nanocomposites.

As evidenced by the low-angle XRD analysis, patterns illustrated in Figure 3A,C,E, mesoscopic order of SBA-15, SBA-16, and KIT-6 porous hosts was well-preserved after Zn addition, since all the characteristic peaks associated with the pristine pore structure symmetries could be clearly detected. Moreover, the absence of peaks assigned to a ZnO crystal phase in the wide-angle area, patterns illustrated in Figure 3B,D,F,G, indicates the high-dispersion degree and the nanocrystalline nature of ZnO species.

**Figure 4:**
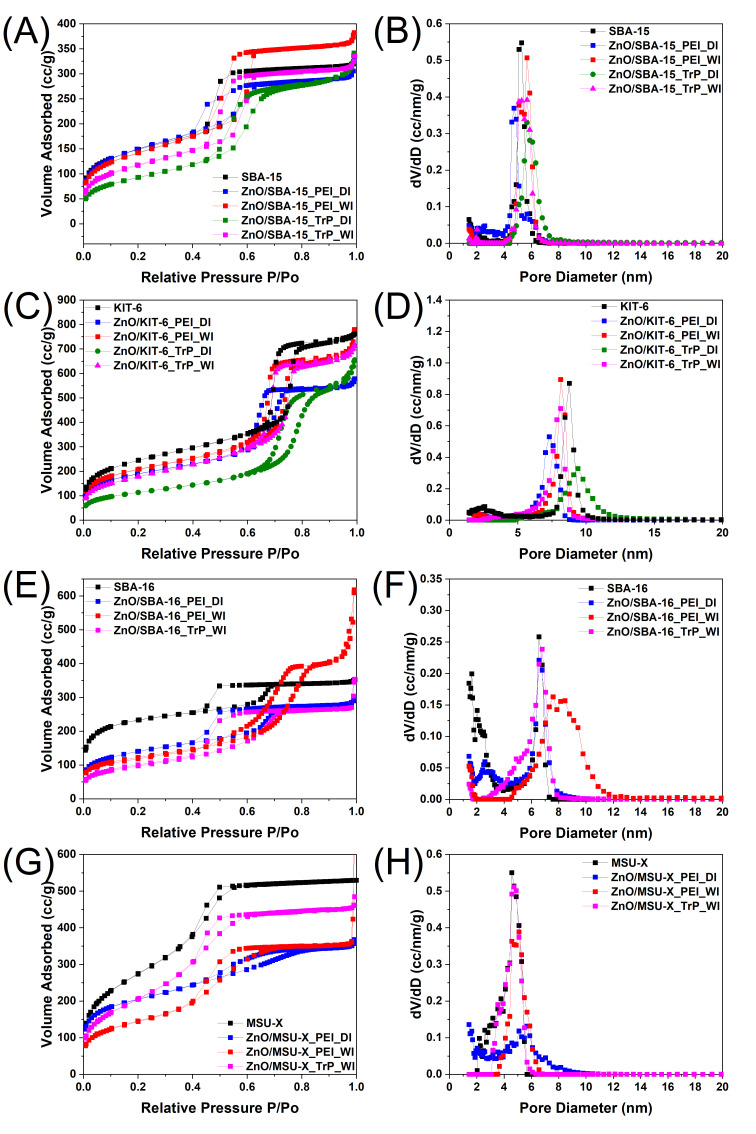
N_2_-physisorption isotherms (**A**,**C**,**E**,**G**) and pore size distribution curves (**B**,**D**,**F**,**H**) of ZnO/SiO_2_ nanocomposites.

According to N_2_ physisorption analysis data, summarized in Table 4 and presented in Figure 4, all the synthesized ZnO/SiO_2_ nanocomposites possess advanced textural features with very high specific surface area and total pore volume values, reaching up to 770 m^2^/g and 1.05 cc/g, respectively. In agreement with XRD results, N_2_ adsorption–desorption isotherms and the corresponding pore size distribution curves further support the preservation of the ordered mesostructures in the final materials. All the isotherms can be classified as type IV featuring a well-defined H1 hysteresis loop. However, Zn sorption modified the pristine network to a different extent, depending on the siliceous pore network, polymer type, and addition process, wet-impregnation vs. dry-impregnation (WI vs. DI). Specifically, the largest shift in both the hysteresis loop and PSD curve was recorded when DI and KIT-6 were employed as the polymer-loading procedure and porous host, respectively. In contrast, the most trivial change was observed when Trilon-P was added through wet impregnation or when SBA-16 was used as host, except for the case of the ZnO/SBA-16_PEI_WI sample.

**Figure 5:**
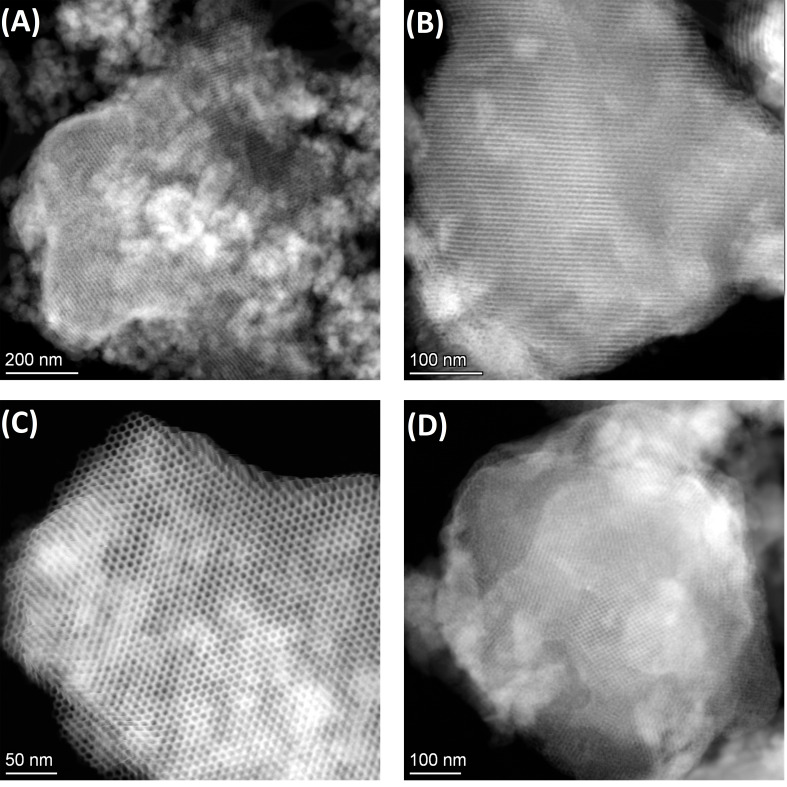
STEM micrographs of (**A**) ZnO/SBA-16_PEI_WI, (**B**) ZnO/SBA-15_PEI_WI, (**C**) ZnO/SBA-15_PEI_DI, and (**D**) ZnO/KIT-6_PEI_DI samples.

To further explore the nanoporous structure and nanocrystalline characteristics of the ZnO NPs, selected samples, i.e., ZnO/SBA-16_PEI_WI, ZnO/SBA-15_PEI_WI, ZnO/SBA-15_PEI_DI, and ZnO/KIT-6_PEI_DI, were examined using TEM microscopy. As shown by the acquired high-angle annular dark field scanning transmission electron microscopy (HAADF-STEM) images, all the investigated nanocomposites exhibit highly homogeneous mesostructures with parallel pore channels in ordered arrays (Figure 5), indicating that Zn introduction did not deteriorate the pristine mesoscopic order, in complete agreement with low angle XRD and N_2_ adsorption analysis. However, in the case of ZnO/SBA-16_PEI_WI sample, a mixed morphology was detected, since monolithic pieces with ordered structure coexist with small amorphous silica particles, accounting for the unusual N_2_ sorption and SEM-EDS findings. Apparently, this sample mixed nature originates from silica’s partial dissolution due to the highly alkaline environment during wet impregnation procedure. Peculiarly, this is the only sample notably affected by the alkaline synthetic conditions, probably due to its irregular pristine morphology.

**Figure 6:**
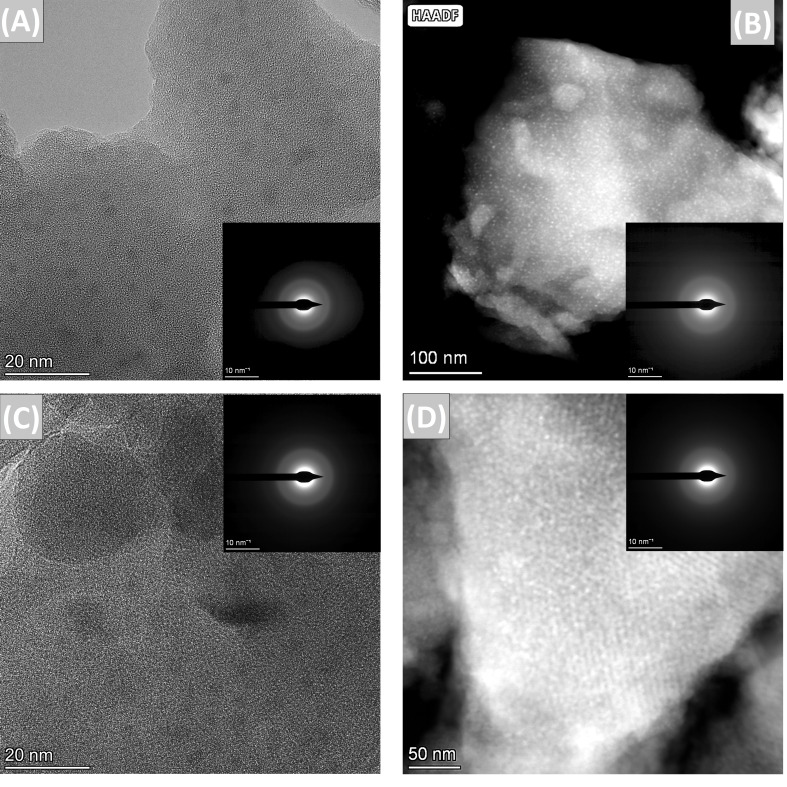
TEM and HAADF-STEM micrographs of (**A**) ZnO/SBA-16_PEI_WI, (**B**) ZnO/SBA-15_PEI_WI, (**C**) ZnO/SBA-15_PEI_DI, and (**D**) ZnO/KIT-6_PEI_DI samples.

**Figure 7:**
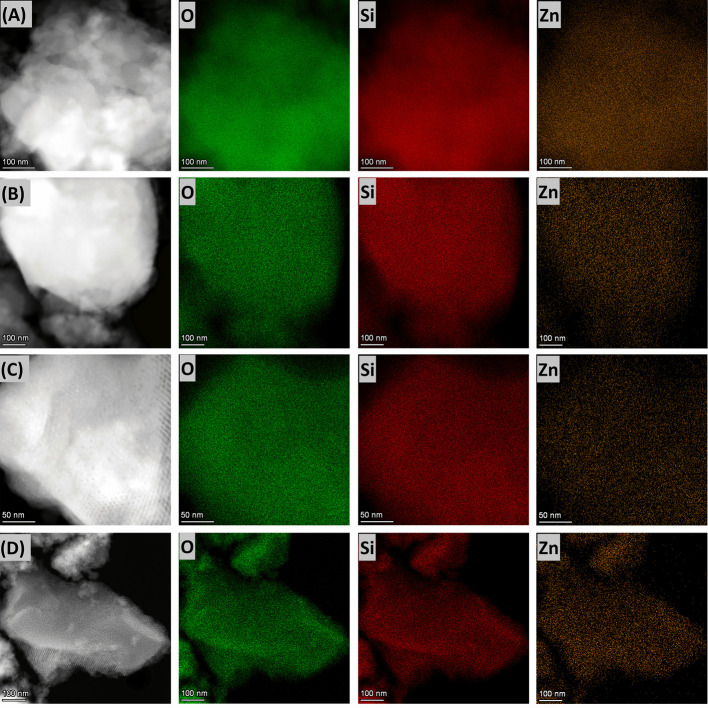
HAADF-STEM micrographs and elemental mappings of (**A**) ZnO/SBA-16_PEI_WI, (**B**) ZnO/SBA-15_PEI_WI, (**C**) ZnO/SBA-15_PEI_DI, and (**D**) ZnO/KIT-6_PEI_DI samples.

In-depth insight into the ZnO particle size, spatial distribution, and dispersion state within the siliceous hosts was obtained using TEM, HAADF-STEM micrographs, selected area diffraction (SAED) patterns, and EDS mapping analysis. As shown in Figure 6, very small ZnO nanoparticles were uniformly dispersed within all silica matrices, confirming the stabilization effect imparted by the proposed methodology. This observation is further corroborated by the EDS mapping analysis, illustrated in Figure 7, revealing a very high compositional uniformity and homogeneous spatial distribution of Zn species at the nanoscale without the detection of Zn-rich nanodomains. Despite the excellent dispersion attained, ZnO NPs size and location differ amongst the samples, evidencing the significant role of the porous host. More precisely, in the ZnO/SBA-16_PEI_WI sample, ultra-small ZnO NPs with a very narrow distribution of 1 to 2 nm were predominantly deposited on the external amorphous silica particles. In contrast, the other samples exhibited a broader size distribution (1–10 nm). Specifically, for ZnO/SBA-15_PEI_WI and ZnO/KIT-6_PEI_DI, a significant fraction of the ZnO NPs was located within the mesoporous channels (Figure 6B,D), whereas in ZnO/SBA-15_PEI_DI, the ZnO NPs were mainly situated at the pore entrances (Figure S37). Finally, the diffuse rings recorded in all the acquired SAED patterns (Figure 6, inserts) revealed the amorphous state of ZnO NPs, in accordance with XRD results. Nevertheless, lattice fringes could be detected in certain nanoparticles by TEM or HRTEM (Figure S37), indicating partial crystallization, which may have been induced by electron beam irradiation.

### 3.4. Antibacterial Activity of ZnO/SiO_2_ Nanocomposites

The antibacterial activity of the as-prepared ZnO-loaded mesoporous silica nanocomposites was assessed against *Staphylococcus aureus* by determining the minimum inhibitory concentration (MIC) and minimum bactericidal concentration (MBC), with a critical focus on the effective Zn concentration (μg/mL Zn), not total composite mass. This Zn-based approach provides a more accurate assessment of antimicrobial efficiency, as it directly reflects the bioavailable antibacterial agent (ZnO) rather than inert support material. The MIC and MBC values were determined followed the Clinical and Laboratory Standards Institute (CLSI) guidelines M07-A9 and M26-A, utilizing the broth macro-dilution and colony-counting methods, respectively [45,46]. The results are summarized in Table 5 and Table 6.

Among all ZnO-loaded mesoporous silica nanocomposites, those employing PEI generally exhibited the lowest MIC and MBC values based on Zn concentrations (Table 5). This superior performance is directly related to the effective ZnO loading and the formation of homogeneously distributed ultrasmall ZnO nanoparticles. Specifically, ZnO/SBA-16_PEI_WI and ZnO/SBA-16_PEI_DI achieved the lowest MIC (0.9 and 0.65 μg/mL Zn, respectively) and MBC (21.6 and 15.6 μg/mL Zn, respectively) values. Despite their modest Zn loading (1.3–1.8 wt.%), their antibacterial activity is remarkable, likely due to the formation of 1–2 nm ZnO NPs deposited on the amorphous silica phase, as well as the presence of highly accessible external ZnO sites and uniform nanoparticle distribution without aggregation, which enhance surface interactions and possibly Zn^2+^ ion release. In case of ZnO/SBA-15_PEI nanocomposites, antibacterial activity was almost comparable to that of ZnO/SBA-16_PEI nanocomposites. Specifically, the sample prepared via the DI method demonstrated slightly stronger antibacterial efficiency (MIC: 1.35 μg/mL Zn, MBC: 24.3 μg/mL Zn) than its WI analog (MIC: 2.6 μg/mL Zn, MBC: 26 μg/mL Zn), despite their similar Zn content (2.7 wt.% DI vs. 2.6 wt.% WI). This performance is likely due to the DI method concentrating ZnO nanoparticles near pore entrances, thereby increasing Zn availability and therefore enhancing surface interactions and possibly Zn^2+^ ion release. For ZnO/MSU-X_PEI nanocomposites, both samples exhibited similar antibacterial performance (MIC: ~4 μg/mL Zn, MBC: ~25 μg/mL Zn), which was slightly lower than that of ZnO/SBA-15_PEI and ZnO/SBA-16_PEI nanocomposites. Interestingly, ZnO/KIT-6_PEI nanocomposites, despite having the highest Zn content coupled with an excellent dispersion (ZnO/KIT-6_PEI_WI 5.9 wt.%, ZnO/KIT-6_PEI_DI 8.1 wt.%), showed the lowest antibacterial efficacy (MIC values 5.9 and 16.2 μg/mL Zn for ZnO/KIT-6_PEI_WI and ZnO/KIT-6_PEI_DI, respectively, and MBC values of 29.5 and 40.5 μg/mL Zn content for ZnO/KIT-6_PEI_WI and ZnO/KIT-6_PEI_DI, respectively). As previously reported (Section 3.3), ZnO NPs in these samples are predominantly confined within the siliceous hosts, thereby limiting their accessibility to bacterial cells as well as potentially retarding the release of Zn^2+^ ions from the siliceous network. Although this uniform dispersion of ZnO NPs within the porous network seems to adversely affect their antibacterial activity, it could be proven highly beneficial, especially at such high metal loading, enhancing materials’ potential for other applications, such as catalysis. On the other hand, ZnO/MSU-X_TrP_WI (MIC: 2 μg/mL Zn, MBC: 5 μg/mL Zn) and ZnO/SBA-15_TrP_DI (MIC: 3 μg/mL Zn, MBC: 5 μg/mL Zn) exhibited the strongest antibacterial activity among the ZnO-loaded siliceous nanocomposites via Trilon-P, as indicated by their lower MIC and MBC values (Table 6). They were followed in effectiveness by ZnO/KIT-6_TrP_DI, ZnO/KIT-6_TrP_WI, ZnO/SBA-15_TrP_WI, and ZnO/SBA-16_TrP_WI.

It should be noted that pure mesoporous silicas, such as SBA-15, SBA-16, MSU-X, and KIT-6, are well recognized as biocompatible materials lacking intrinsic antibacterial activity [52,53,54]. Antimicrobial properties arise only upon suitable surface functionalization [55] or incorporation of active nanoparticles, such as ZnO NPs [36,37,54] or active compounds [56,57,58]. On the other hand, it is well established that ZnO NPs exhibited strong antibacterial properties. The MIC values reported for bare ZnO NPs vary considerably, typically ranging from 10 to 1000 μg/mL, depending on particle size, morphology, surface area-to-volume ratio, synthesis route, and the bacterial strain tested [13,17,21,48,59,60,61,62]. For example, in our previous publication, ZnO NPs with a polyhedral plate-like morphology and an average particle size of 28 nm exhibited pronounced antibacterial activity against the same *S. aureus* strain, with MIC and MBC values of 20 and 50 μg/mL, respectively [48]. In another study, spherical ZnO nanoparticles with an average diameter of ~7 nm exhibited inhibitory activity against *S. aureus* at a concentration of 250 μg/mL, whereas larger particles (~150 nm) required concentrations up to 1000 μg/mL to achieve comparable antibacterial effects [19]. Immobilization of ZnO NPs on silica supports has been reported to enhance antibacterial performance [27,36,37]. Shehata et al. reported that silver/zinc-incorporated mesoporous silica nanoparticles (MCM-48) exhibited strong antibacterial activity against *S. aureus*, *E. coli*, and methicillin-resistant *S. aureus* (MRSA), with MICs ranging from 7.8 to 62.5 μg/mL [63]. Likewise, ZnO nanoparticles supported on mesoporous silica SBA-3 inhibited the growth of *E. coli* and *S. aureus*, displaying MIC values of 1.24 and 0.31 mg/mL, respectively [36]. More recently, Trinh et al. demonstrated that Zn-loaded mesoporous silicas (SBA-1 and SBA-15) showed pronounced antibacterial activity against *E. coli* and *Bacillus subtilis* at a concentration of 20 μg/mL [54]. Notably, in the present study, superior antibacterial activity was achieved, with comparable inhibition reached at substantially lower concentrations (MICs 1–2.5 μg/mL Zn content). Specifically, the optimum antibacterial performance was achieved by ZnO NPs loaded on SBA-15 and SBA-16 via PEI regardless of the loading method as well as by ZnO NPs loaded on MSU-X via TrP (MICs 1–2.5 μg/mL Zn). These findings underscore the key role of the siliceous host properties, particularly morphology and pore architecture. In SBA-16 and MSU-X samples, ZnO NPs are finely dispersed either on or near the external siliceous surface as a result of the narrow pore entrances in the former and the small-sized mesopores in the latter, which impede the penetration of the larger Trilon-P molecule. These features probably enhance the interactions of ZnO NPs with the bacterial cell envelope and promote the release of Zn^2+^ ions. In SBA-15, the plate-like morphology generates short mesochannels with abundant pore openings, yielding a high population of exposed ZnO NPs. In contrast, the open three-dimensional pore network of KIT-6 facilitates the confinement of ZnO NPs, but in combination with its dense monolithic morphology, this restricts NPs accessibility and thereby limits their interaction with the bacteria and probably retards the release of Zn^2+^ ions. Thus, bioactivity appears to depend more on nanoparticle accessibility, dispersion, and location than on total Zn loading, highlighting the importance of balancing ZnO content with suitable pore structure and diffusion efficiency.

**Figure 8:**
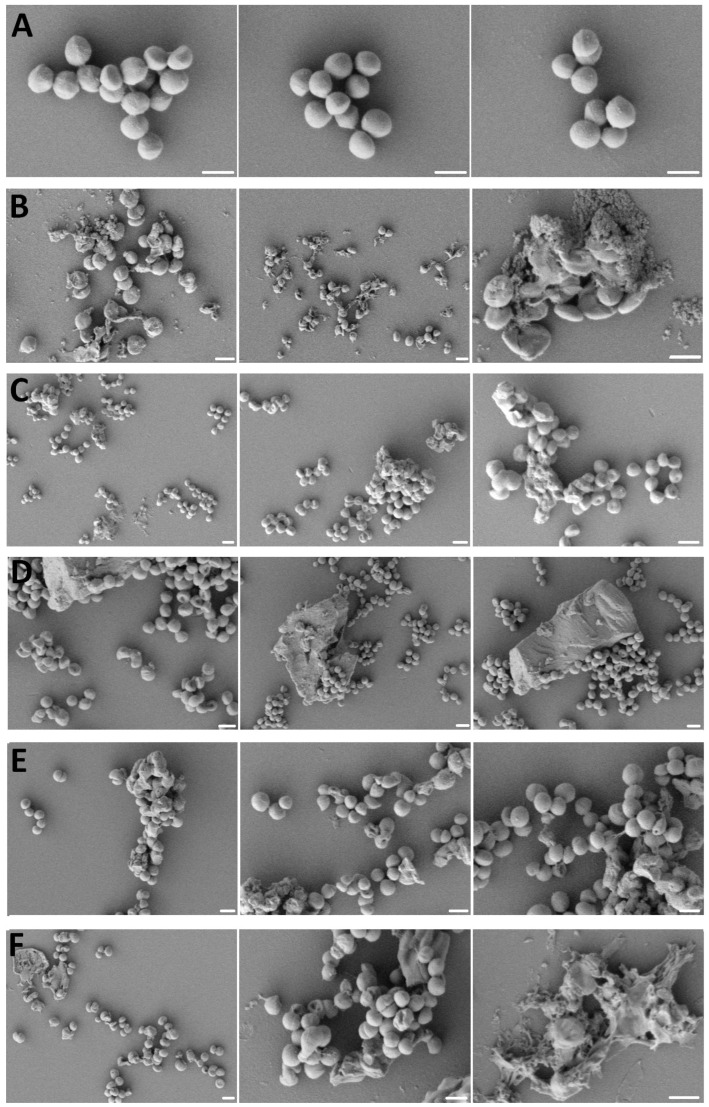
SEM images of *S. aureus* bacteria: (**A**) untreated cells (control) and cells after treatment with (**B**) ZnO/SBA-16_PEI_WI, (**C**) ZnO/SBA-15_PEI_WI, (**D**) ZnO/SBA-16_PEI_DI, (**E**) ZnO/SBA-15_PEI_DI, and (**F**) ZnO/MSU-X_TrP_WI for 12 h at their MIC. The scale bar in each micrograph corresponds to 1 μm.

The morphology of *S. aureus* after treatment was examined by scanning electron microscopy (SEM). Bacterial cells were exposed to the most active ZnO/mesoporous silica nanocomposites at MIC for 12 h, and changes in the surface morphology and membrane structure were evaluated. Figure 8 shows SEM images of untreated cells (control) and those treated with the nanocomposites. The untreated bacteria (Figure 8A) display a typical spherical morphology with intact cell walls, whereas cells treated with all ZnO-loaded nanocomposites (Figure 8B–F) exhibit pronounced morphological alterations. Treated cells appear wrinkled and structurally damaged, with evidence of intracellular content leakage, deformation, and extensive cell death. These observations are consistent with previous reports, which describe ZnO NPs as initially disrupting bacterial membranes and cell walls, followed by leakage of intracellular components and eventual cell death. The antibacterial mechanism of ZnO nanoparticles (ZnO NPs) is not yet fully understood [64,65,66], although several complementary pathways have been proposed. These include (a) direct interaction of ZnO NPs with bacteria cell walls, leading to membrane disruption and subsequent internalization either through the cell envelope or via endocytic-like processes; (b) generation of reactive oxygen species (ROS), which induce oxidative stress and inhibit DNA replication and protein synthesis [64]; and (c) release of Zn^2+^ ions upon ZnO dissolution, leading to membrane integrity loss, collapse of the proton motive force, and blockage of ion channels [67]. In a related study, Trinh et al. demonstrated that the antibacterial activity of Zn-loaded mesoporous silicas (SBA-1 and SBA-15) against *E. coli* and Bacillus subtilis was primarily attributed to their strong interaction with bacterial cell walls and the sustained release of Zn^2+^ ions [54]. In line with these findings, the ZnO/mesoporous silica nanocomposites investigated in this study likely exert their antibacterial action through a synergistic interplay of physical and chemical mechanisms, involving interaction with the bacterial cell envelope, release of Zn^2+^ ions, and ROS generation, ultimately leading to membrane destabilization, impairment of metabolic and enzymatic functions, and bacterial cell death.

### 3.5. In Vitro Cytotoxicity Evaluation of ZnO/SiO_2_ Nanocomposites

The widespread application of ZnO NPs in the pharmaceutical, cosmetic, textile, and agricultural sectors has raised increasing concerns regarding their potential toxicological impacts on both the environment and human health. ZnO NPs are known to induce cytotoxicity primarily through the release of Zn^2+^ ions and the generation of reactive oxygen species (ROS), which together trigger oxidative stress, mitochondrial dysfunction, DNA damage, and ultimately cell death [68,69]. Among these mechanisms, Zn^2+^ dissolution is generally regarded as the predominant contributor to cytotoxicity, while intracellular ROS formation amplifies the oxidative imbalance and accelerates apoptotic pathways [70,71]. The magnitude of these effects is strongly influenced by the physicochemical characteristics of the nanoparticles—particularly their size, morphology, and surface chemistry [19,68]. Smaller (<50 nm) and anisotropic ZnO nanostructures, such as nanorods, display enhanced reactivity and faster Zn^2+^ release due to their high surface-to-volume ratio, resulting in stronger cytotoxic responses compared with spherical or microscale particles [19].

In view of these findings, systematic evaluation of the cytotoxicity of ZnO-based materials is essential prior to their practical use. In the present study, the cytotoxic effects of the as-prepared ZnO-loaded mesoporous silica nanocomposites were investigated at MIC-related concentrations. Specifically, the in vitro cytotoxicity was assessed using HEK293 (normal human embryonic kidney) and PC3 (prostate cancer) cell lines via the MTT assay following 24 h of exposure.

**Figure 9:**
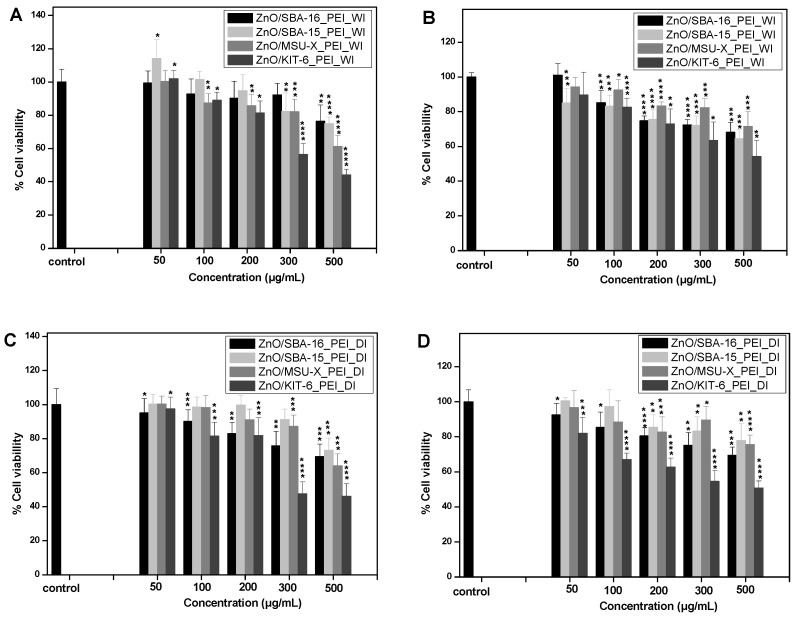
Comparative cytotoxicity of ZnO-loaded mesoporous silica nanocomposites via PEI, using either wet impregnation (WI) or dry impregnation (DI), on HEK293 (**A**,**C**) and PC3 (**B**,**D**) cells, assessed by MTT assay after 24 h incubation. Results are presented as mean ± SD from six values obtained in at least three independent experiments. Statistical significance (Student’s paired two-tailed *t*-test) is denoted as * *p* < 0.05, ** *p* < 0.01, *** *p* < 0.001, and **** *p* < 0.0001; absence of annotation indicates no significance (*p* > 0.05).

In the case of ZnO NPs loaded on mesoporous silicas via PEI, cell viability decreased in a dose-dependent manner (Figure 9 and Figure S38). Specifically, both HEK293 and PC3 cells treated with ZnO NPs loaded on SBA-15, SBA-16, or MSU-X via PEI using WI-loading method, maintained relatively high viability (>80%) at concentration up to 300 μg/mL (corresponding to 7.8, 5.4, and 12.9 μg/mL Zn content, respectively), while at 500 μg/mL (corresponding to 13, 9, and 21.5 μg/mL Zn content, respectively), cell viability was slightly reduced to 60–70%. On the other hand, the DI-prepared counterparts exhibited similar cytotoxicity towards HEK293 cells, whereas in PC3 cells, these nanocomposites were found to be less toxic (cell viability: >75% at 500 μg/mL, corresponding to 6.5–21 μg/mL Zn content). It should be noted that all these nanocomposites, regardless of the loading method, were non-toxic to both tested cell lines at MIC-related concentrations (cell viability ≥ 90% at their MIC; Figure 9 and Figure S38). Among them, ZnO/SBA-16_PEI_WI and ZnO/SBA-15_PEI_DI exhibited negligible toxicity, showing nearly 100% cell viability at their MIC (50 μg/mL, corresponding to 0.9 and 1.35 μg/mL Zn content, respectively; Table 5 and Figure 9 and Figure S38). ZnO NPs loaded on KIT-6 via PEI, regardless of the loading method, were non-toxic only at low concentrations, close to their MIC (survival: >80% at 100–200 μg/mL, corresponding to 5.9–16.2 μg/mL Zn content). However, at higher concentrations, HEK293 cell viability was further reduced, dropping below 60%, while PC3 cells retained slightly higher viability at equivalent doses. This higher toxicity of both ZnO/KIT-6_PEI nanocomposites compared to the others may be attributed to their higher Zn content (ZnO/KIT-6_PEI_WI 5.9 wt.%, ZnO/KIT-6_PEI_DI 8.1 wt.%).

**Figure 10:**
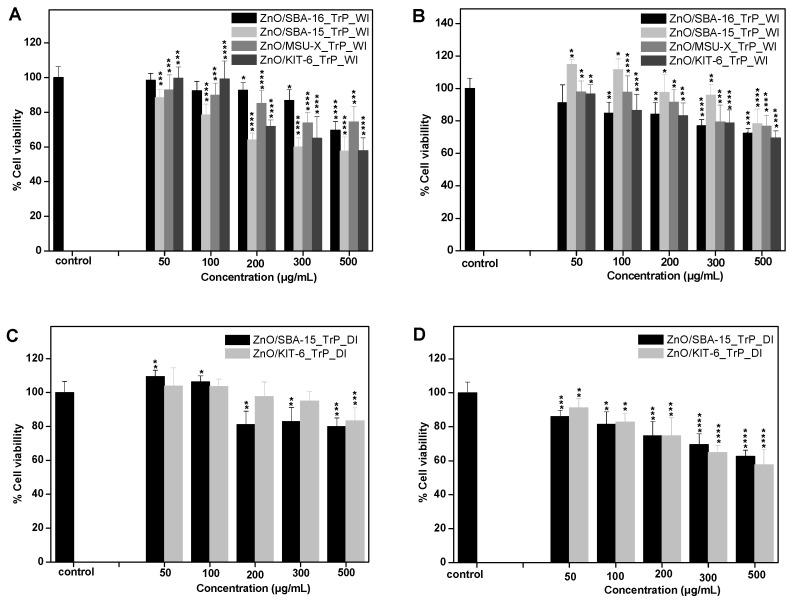
Comparative cytotoxicity of ZnO-loaded mesoporous silica nanocomposites via TrP, using either wet impregnation (WI) or dry impregnation (DI), on HEK293 (**A**,**C**) and PC3 (**B**,**D**) cells, assessed via MTT assay after 24 h incubation. Results are presented as mean ± SD from six values obtained in at least three independent experiments. Statistical significance (Student’s paired two-tailed *t*-test) is denoted as * *p* < 0.05, ** *p* < 0.01, *** *p* < 0.001, and **** *p* < 0.0001; absence of annotation indicates no significance (*p* > 0.05).

For ZnO NPs loaded on mesoporous silicas via TrP (Figure 10 and Figure S39), cytotoxic effects were generally milder in both cell lines compared to PEI analogs. Particularly, the WI-prepared nanocomposites induced only moderate reductions in PC3 cell viability (75–80% at 500 μg/mL). In the case of HEK293, ZnO/SBA-16_PEI_WI and ZnO/MSU-X_PEI_WI retained higher viability (>70% at 500 μg/mL) compared to the other two nanocomposites. On the other hand, the DI-prepared nanocomposites were essentially non-toxic to HEK293 cells even at the higher tested concentration, whereas in PC3 cells, they demonstrated slightly stronger cytotoxic effects, with viability dropping to ~60% at 500 μg/mL. However, these nanocomposites exhibited a slightly toxicity in both tested cell lines at their MIC (cell survival: 70–90%), except ZnO/MSU-X_TrP_WI, which did not exhibit any toxicity at its MIC (cell viability 85–90% at 200 μg/mL, corresponding to 2 μg/mL Zn content; Table 6 and Figure 10 and Figure S39).

Taken together, the results demonstrate that the cytotoxicity of ZnO/mesoporous silica nanocomposites is mainly influenced by the type of mesoporous silica host, while the loading method and polymer type exert comparatively minor effects. Correlation of the antibacterial and cytotoxicity data further reveals that the nanocomposites exhibiting the most effective antibacterial performance—namely, ZnO NPs loaded on SBA-15 and SBA-16 via PEI (both WI and DI) as well as ZnO NPs loaded on MSU-X via TrP employing WI—did not display significant cytotoxicity (cell survival > 90%) at MIC-related concentrations (50–100 μg/mL corresponding to 0.65–2 μg/mL Zn content).

The improved biocompatibility of these nanocomposites can be attributed to the protective effect of the mesoporous silica framework, which acts as a physical barrier that regulates Zn^2+^ ion dissolution and limits direct contact between ZnO and cellular components. The ZnO incorporation into siliceous hosts effectively attenuates ROS-mediated oxidative damage, while preserving the inherent antimicrobial performance of ZnO. These observations are consistent with other reports indicating that silica coating or integration of ZnO within mesoporous matrices effectively mitigates ZnO toxicity by suppressing ion leaching and surface reactivity, thereby achieving an optimal balance between antibacterial efficiency and cellular safety [63,72,73,74].

Overall, the present findings establish ZnO-loaded SBA-15, SBA-16, and MSU-X systems as promising safer-by-design candidates that combine potent antibacterial activity with minimal cytotoxicity, rendering them suitable for future biomedical and antimicrobial applications.

### 3.6. Life Cycle Assessment of Selected ZnO/SiO_2_ Nanocomposites

**Figure 11:**
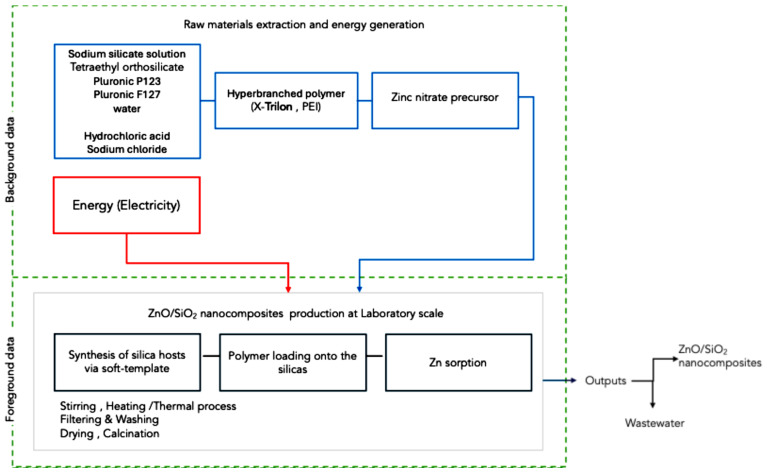
System boundaries for the evaluated ZnO/SiO_2_ nanocomposites at the laboratory scale.

The environmental impacts associated with the laboratory-scale production of the ZnO/SiO_2_ nanocomposites exhibiting the best antibacterial performance were quantitatively assessed through a cradle-to-gate system Life Cycle Assessment, in accordance with the ISO 14040:2006 framework [75,76]. The defined system boundaries encompassed all stages from raw material extraction to nanocomposite synthesis (Figure 11), thereby representing a cradle-to-gate assessment. The use and end-of-life phases were excluded due to the lack of reliable data on nanomaterial release mechanisms and disposal pathways, a common limitation in current LCAs of nanomaterials. These boundaries and the corresponding life cycle inventory were developed from primary experimental data. The inventory captures the specific material and energy flows associated with the background and foreground data and forms the basis for the subsequent environmental impact assessment. Results are expressed per 1 g of nanocomposite, corresponding to the functional unit defined in Section 2.7.

#### 3.6.1. Life Cycle Assessment Results

**Figure 12:**
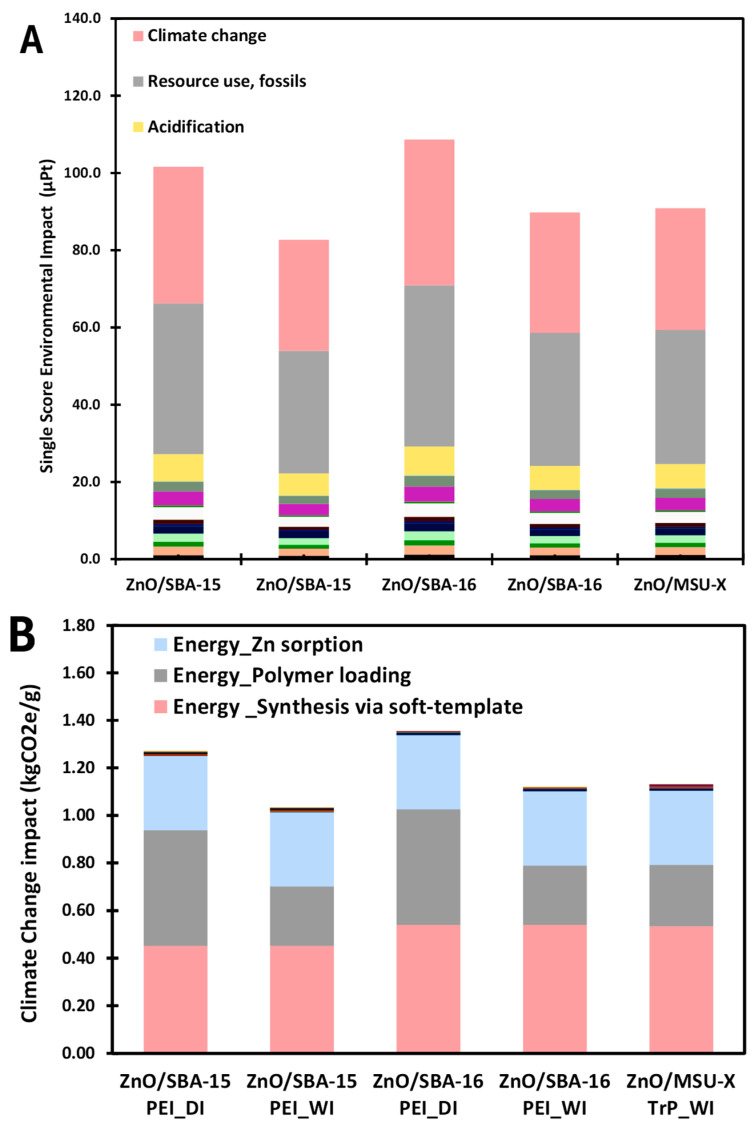
(**A**) Comparative environmental impact (single score). (**B**) Breakdown of climate change contributions of ZnO-loaded mesoporous silica nanocomposites with the best antibacterial performance.

Figure 12A presents the total environmental impacts, expressed in micropoints. These results reflect the environmental burdens linked to production processes only and do not capture potential impacts or benefits arising during product application or disposal. Environmental impact variations arise mainly from the energy demand associated with the preparation of silica supports under different time and temperature conditions (stirring, thermal treatment, and drying), as well as the use of polymers and reagents. The categories of climate change, fossil resource use, and acidification dominate, together accounting for more than 80% of the total score.

The climate change analysis was carried out for a series of ZnO-loaded mesoporous silica nanocomposites synthesized through a three-step protocol. Each stage of the synthesis was found to contribute differently to the overall impact, with energy demand being the predominant driver (Figure 12B). In the first step, involving the soft-templated synthesis of mesostructured siliceous hosts, SBA-15 exhibited the lowest energy consumption, while SBA-16 and MSU-X required comparatively higher inputs due to differences in processing conditions. In the second step, corresponding to polymer loading, wet impregnation consistently minimized the energy demand relative to dry impregnation, particularly for polyethyleneimine (PEI), where the vacuum-drying stage in the dry route imposed an additional energy burden. The third step, Zn sorption onto the polymer-modified supports, was identical for all nanocomposites and hence provided a similar contribution.

Across all examined nanocomposites, the climate change values ranged from 1.00 to 1.36 kg CO_2_e per gram. Based on these results, ZnO/SBA-15_PEI_WI exhibited the lowest climate change impact among the evaluated nanocomposites, while ZnO/SBA-16_PEI_DI presented the highest, followed by ZnO/SBA-15_PEI_DI, ZnO/SBA-16_PEI_WI, and ZnO/MSU-X_TrP_WI. The latter reflected the additional energy required for TrP synthesis from PEI and the inherently higher environmental burden of TrP compared to PEI.

For reference, the recent literature reports net emissions for SBA-16 and SBA-15 mesoporous silica synthesis in the range of 0.82 to 1.939 kg CO_2_ eq. per gram at laboratory scale (cradle-to-gate boundary) [77]. These values serve as preliminary baseline references, as there is currently no available data in the literature for commercial nanocomposites or directly comparable materials with identical compositions. Therefore, this comparison should be considered indicative rather than definitive.

#### 3.6.2. Life Cycle Assessment Limitations

Several limitations should be considered when interpreting these results. Laboratory-scale synthesis inherently exhibits low throughput and operational inefficiencies, leading to higher energy intensity per functional unit, particularly in energy-intensive categories, such as climate change and fossil resource depletion. Scaling up to pilot or industrial production is expected to improve process efficiency through enhanced heat and mass transfer, more stable operational control, and reduced idle energy use, resulting in more representative life cycle inventories.

Conventional LCA databases also largely lack characterization factors for nanomaterial-specific emissions, transformations, and toxicity, which constrains the assessment of potential risks associated with nanoscale releases. In addition, an uncertainty analysis of input parameters, such as Monte Carlo simulations, was not performed and is recommended for future studies to provide a more rigorous evaluation. Finally, future research should explore the integration of renewable energy sources and process intensification strategies to reduce environmental impacts and enhance the overall sustainability of the impregnation-based synthesis routes.

## 4. Conclusions

In this study, a dendritic polymer-assisted templating approach was developed to deliver ultrasmall ZnO nanoparticles stabilized within different mesoporous silica hosts (SBA-15, SBA-16, KIT-6, and MSU-X) as effective antibacterial systems. Hyperbranched polyethyleneimine (PEI) and carboxy-methylated polyethyleneimine (Trilon-P, TrP) were employed as metal-trapping and -templating agents via both wet (WI) and dry impregnation (DI) methods, enabling precise control over ZnO nanoparticle loading and distribution. Low-angle XRD confirmed that the mesoscopic order of SBA-15, SBA-16, and KIT-6 was preserved after ZnO loading, while wide-angle XRD evidenced the nanocrystalline and highly dispersed nature of ZnO. N_2_ adsorption–desorption isotherms and pore size distribution analyses demonstrated advanced textural properties (surface areas up to 770 m^2^/g and pore volumes up to 1.05 cm^3^/g), with polymer functionalization and ZnO loading inducing host-dependent modifications. SEM-EDS mapping revealed homogeneous Zn distribution across the silica frameworks without external aggregation, while TEM and HAADF-STEM imaging confirmed the formation of finely dispersed ultrasmall ZnO nanoparticles (1–10 nm), with their location—within mesoporous channels or at the external surface/pore entrances—being strongly dependent on the siliceous host. Biological assays demonstrated that the nanocomposites combined high antibacterial activity with low cytotoxicity. ZnO NPs loaded on SBA-15 and SBA-16 via PEI (both WI and DI), as well as ZnO NPs loaded on MSU-X via TrP employing WI, achieved the lowest MIC values (1–2.5 μg/mL Zn) against *S. aureus*, outperforming bare ZnO nanoparticles. Cytotoxicity studies in HEK293 and PC3 cells revealed that these nanocomposites maintained >90% survival at antibacterial MIC-related concentrations, indicating excellent biocompatibility. The LCA results indicate that ZnO/SiO_2_ nanocomposites are most affected by energy-intensive synthesis steps, with climate change, fossil resource use, and acidification representing the dominant impact categories. Among the evaluated materials, ZnO/SBA-15_PEI_WI showed the lowest environmental burden, while ZnO/SBA-16_PEI_DI exhibited the highest. Overall, the findings highlight the dominant role of the siliceous host pore geometry in controlling ZnO nanoparticle spatial distribution and biological performance, while the hyperbranched polymer type, as well as the loading method, act as secondary modulators. Additionally, the LCA results confirm that synthesis conditions and polymer choice directly influence the overall environmental footprint of the nanocomposites. The proposed polymer-assisted templating strategy offers a versatile route to engineer ZnO/mesoporous silica nanocomposites with tunable sustainability, antibacterial potency, and minimal cytotoxicity, making them promising candidates for next-generation antibacterial systems.

## Figures and Tables

**Table 1:** Functional unit and the systems introduced in SimaPro software.

Functional Unit	1 g of Synthesized ZnO/SiO_2_
**Method**	EF3.1 (adapted)
**Geography**	Europe
**Dataset**	Ecoinvent v3.11
**Data type**	Primary data (experimental cases at laboratory scale under the scope of this work)

**Table 2:** Pore structural properties of pristine mesoporous silicas.

Samples	SSA (m^2^ g^−1^)	TPV (cc g^−1^)	Average Pore Diameter (nm)
**SBA-15**	546	0.49	3.6
**KIT-6**	876	1.16	5.3
**SBA-16**	871	0.53	2.4
**MSU-X**	1001	0.82	3.3

**Table 3:** Pore structural properties of organically modified mesoporous silicas with PEI, and Trilon-P (TrP) via wet impregnation (WI) and dry impregnation (DI), along with polymer content derived via TGA analysis.

Samples	SSA (m^2^ g^−1^)	TPV (cc g^−1^)	Average Pore Diameter (nm)	Polymer Content (wt.%)
**SBA-15_PEI_WI**	223	0.28	5	17
**KIT-6_PEI_WI**	297	0.57	7.7	27
**SBA-16_PEI_WI**	191	0.39	8.3	21
**MSU-X_PEI_WI**	432	0.38	3.5	22
**SBA-15_PEI_DI**	33.2	0.046	5.6	24
**KIT-6_PEI_DI**	277	0.46	6.7	30
**SBA-16_PEI_DI**	359	0.34	3.8	9
**MSU-X_PEI_DI**	94	0.12	5.3	25
**SBA-15_TrP_WI**	436	0.47	4.3	5.5
**KIT-6_TrP_WI**	600	0.96	6.4	11
**SBA-16_TrP_WI**	534	0.45	3.3	6
**MSU-X_TrP_WI**	705	0.65	3.7	7.5
**SBA-15_TrP_DI**	333	0.45	5.4	5
**KIT-6_TrP_DI**	391	0.83	8.4	9

**Table 4:** Zn content in final nanocomposites, as well as the pore structural properties of ZnO/SiO_2_ nanocomposites.

Samples	Zn Loading (wt.%)	SSA (m^2^ g^−1^)	TPV (cc g^−1^)	Average Pore Diameter (nm)
**ZnO/SBA-15_PEI_WI**	2.6	511	0.56	4.4
**ZnO/KIT-6_PEI_WI**	5.9	742	1.05	5.7
**ZnO/SBA-16_PEI_WI**	1.8	442	0.67	6.0
**ZnO/MSU-X_PEI_WI**	4.3	519	0.55	4.2
**ZnO/SBA-15_PEI_DI**	2.7	535	0.45	3.4
**ZnO/KIT-6_PEI_DI**	8.1	683	0.85	5.0
**ZnO/SBA-16_PEI_DI**	1.3	507	0.43	3.4
**ZnO/MSU-X_PEI_DI**	4.2	742	0.54	2.9
**ZnO/SBA-15_TrP_WI**	0.9	420	0.48	4.6
**ZnO/KIT-6_TrP_WI**	1.8	644	1.04	6.4
**ZnO/SBA-16_TrP_WI**	0.75	350	0.41	4.7
**ZnO/MSU-X_TrP_WI**	1.0	769	0.70	3.6
**ZnO/SBA-15_TrP_DI**	1.0	335	0.47	5.6
**ZnO/KIT-6_TrP_DI**	0.9	411	0.88	8.6

**Table 5:** MIC and MBC values of ZnO-loaded siliceous nanocomposites via PEI, employing either wet impregnation (WI) or dry impregnation (DI) against *S. aureus* bacteria. Both total concentrations and the corresponding Zn actual concentrations are shown for all nanocomposites.

Samples		MIC (μg/mL)	MBC (μg/mL)
**ZnO/SBA-16_PEI_WI**	Total	50	1200
	Zn content	0.9	21.6
**ZnO/SBA-15_PEI_WI**	Total	100	1000
	Zn content	2.6	26.0
**ZnO/MSU-X_PEI_WI**	Total	100	600
	Zn content	4.3	25.8
**ZnO/KIT-6_PEI_WI**	Total	100	500
	Zn content	5.9	29.5
**ZnO/SBA-16_PEI_DI**	Total	50	1200
	Zn content	0.65	15.6
**ZnO/SBA-15_PEI_DI**	Total	50	900
	Zn content	1.35	24.3
**ZnO/MSU-X_PEI_DI**	Total	100	600
	Zn content	4.2	25.3
**ZnO/KIT-6_PEI_DI**	Total	200	500
	Zn content	16.2	40.5

**Table 6:** MIC and MBC values of ZnO-loaded siliceous nanocomposites via Trilon-P (TrP), employing either wet impregnation (WI) or dry impregnation (DI) against *S. aureus* bacteria. Both total concentrations and the corresponding Zn actual concentrations are shown for all nanocomposites.

Samples		MIC (μg/mL)	MBC (μg/mL)
**ZnO/SBA-16_TrP_WI**	Total	400	1500
	Zn content	3.0	11.25
**ZnO/SBA-15_TrP_WI**	Total	400	1200
	Zn content	3.6	10.8
**ZnO/MSU-X_TrP_WI**	Total	200	500
	Zn content	2.0	5.0
**ZnO/KIT-6_TrP_WI**	Total	200	600
	Zn content	3.6	10.8
**ZnO/SBA-15_TrP_DI**	Total	300	500
	Zn content	3.0	5.0
**ZnO/KIT-6_TrP_DI**	Total	400	900
	Zn content	3.6	8.1

## Data Availability

The original contributions presented in this study are included in the article/Supplementary Material. Further inquiries can be directed to the corresponding authors.

## Supplementary Materials

The following supporting information can be downloaded at https://www.mdpi.com/article/10.3390/nano15221697/s1: Figure S1: Wide (A) and Low angle XRD patterns (B) of pristine mesoporous silica samples; Figure S2: N_2_-physisorption isotherms (A) and Pore Size Distribution Curves (B) of pure silicas; Figure S3: TGA profiles of SBA-15 sample loaded with PEI through wet impregnation at different pH values; Figure S4: TGA profiles of mesoporous silicas loaded with PEI via wet impregnation; Figure S5: TGA profiles of mesoporous silicas loaded with PEI via dry impregnation; Figure S6: N_2_-physisorption isotherms (A) and Pore Size Distribution Curves (B) of pure SBA-15 and PEI-loaded SBA-15 (SBA15_PEI_WI) via wet impregnation; Figure S7: N_2_-physisorption isotherms (A) and Pore Size Distribution Curves (B) of pure KIT-6 and PEI-loaded KIT-6 via wet impregnation (KIT-6_PEI_WI); Figure S8: N_2_-physisorption isotherms (A) and Pore Size Distribution Curves (B) of pure SBA-16 and PEI-loaded SBA-16 via wet impregnation (SBA-16_PEI_WI); Figure S9: N_2_-physisorption isotherms (A) and Pore Size Distribution Curves (B) of pure MSU-X and PEI-loaded MSU-X via wet impregnation (MSU-X_PEI_WI); Figure S10: N_2_-physisorption isotherms (A) and Pore Size Distribution Curves (B) of pure SBA-15 and PEI-loaded SBA-15 via dry impregnation (SBA-15_PEI_DI); Figure S11: N_2_-physisorption isotherms (A) and Pore Size Distribution Curves (B) of pure KIT-6 and PEI-loaded KIT-6 via dry impregnation (KIT-6_PEI_DI); Figure S12: N_2_-physisorption isotherms (A) and Pore Size Distribution Curves (B) of pure SBA-16 and PEI-loaded SBA-16 via dry impregnation (SBA-16_PEI_DI); Figure S13: N_2_-physisorption isotherms (A) and Pore Size Distribution Curves (B) of pure MSU-X and PEI-loaded MSU-X via dry impregnation (MSU-X_PEI_DI); Figure S14: TGA profiles of mesoporous silicas loaded with Trilon-P (TrP) via wet impregnation; Figure S15: TGA profiles of mesoporous silicas loaded with Trilon-P via dry impregnation; Figure S16: TGA profiles of SBA-15 sample loaded with Trilon-P (TrP) through wet impregnation at different pH values; Figure S17: N_2_-physisorption isotherms (A) and Pore Size Distribution Curves (B) of pure SBA-15 and Trilon-P-loaded SBA-15 via wet impregnation (SBA-15_TrP_WI); Figure S18: N_2_-physisorption isotherms (A) and Pore Size Distribution Curves (B) of pure KIT-6 and Trilon-P-loaded KIT-6 via wet impregnation (KIT-6_TrP_WI); Figure S19: N_2_-physisorption isotherms (A) and Pore Size Distribution Curves (B) of pure SBA-16 and Trilon-P-loaded SBA-16 via wet impregnation (SBA-16_TrP_WI); Figure S20: N_2_-physisorption isotherms (A) and Pore Size Distribution Curves (B) of pure MSU-X and Trilon-P-loaded MSU-X via wet impregnation (MSU-X_TrP_WI); Figure S21: N_2_-physisorption isotherms (A) and Pore Size Distribution Curves (B) of pure SBA-15 and Trilon-P-loaded SBA-15 via dry impregnation (SBA-15_TrP_DI); Figure S22. N_2_-physisorption isotherms (A) and Pore Size Distribution Curves (B) of pure KIT-6 and Trilon-P loaded KIT-6 via dry impregnation (KIT-6_TrP_DI); Figure S23. TGA profile of the dried ZnO/SBA-15_PEI_DI; Figure S24: (A) SEM micrograph of ZnO/SBA-15_PEI_DI and the corresponding (B) O, (C) Si and (D) Zn elemental maps; Figure S25: (A) SEM micrograph of ZnO/SBA-16_PEI_DI and the corresponding (B) O, (C) Si and (D) Zn elemental maps; Figure S26: (A) SEM micrograph of ZnO/MSU-X_PEI_DI and the corresponding (B) O, (C) Si and (D) Zn elemental maps; Figure S27: (A) SEM micrograph of ZnO/SBA-15_PEI_WI sample and the corresponding (B) O, (C) Si and (D) Zn elemental maps; Figure S28: (A) SEM micrograph of ZnO/KIT-6_PEI_WI sample and the corresponding (B) O, (C) Si and (D) Zn elemental maps; Figure S29: (A) SEM micrograph of ZnO/SBA-16_PEI_WI sample and the corresponding (B) O, (C) Si and (D) Zn elemental maps; Figure S30: (A) SEM micrograph of ZnO/MSU-X_PEI_WI sample and the corresponding (B) O, (C) Si and (D) Zn elemental maps; Figure S31: (A) SEM micrograph of ZnO/SBA-15_TrP_WI sample and the corresponding (B) O, (C) Si and (D) Zn elemental maps; Figure S32: (A) SEM micrograph of ZnO/KIT-6_TrP_WI sample and the corresponding (B) O, (C) Si and (D) Zn elemental maps; Figure S33: (A) SEM micrograph of ZnO/SBA-16_TrP_WI sample and the corresponding (B) O, (C) Si and (D) Zn elemental maps; Figure S34: (A) SEM micrograph of ZnO/MSU-X_Trilon-P_WI sample and the corresponding (B) O, (C) Si and (D) Zn elemental maps; Figure S35: (A) SEM micrograph of ZnO/SBA-15_TrP_DI sample and the corresponding (B) O, (C) Si and (D) Zn elemental maps; Figure S36: (A) SEM micrograph of ZnO/KIT-6_TrP_DI sample and the corresponding (B) O, (C) Si and (D) Zn elemental maps; Figure S37: TEM micrographs of ZnO/SBA-15_PEI_DI sample; Figure S38. Comparative cytotoxicity of ZnO-loaded mesoporous silica nanocomposites via PEI, using either wet impregnation (WI) or dry impregnation (DI), on HEK293 (A,C) and PC3 (B,D) cells as a function of Zn content, assessed by MTT assay after 24 h incubation. Results are presented as mean ± SD from six values obtained in at least three independent experiments; Figure S39. Comparative cytotoxicity of ZnO-loaded mesoporous silica nanocomposites via TrP, using either wet impregnation (WI) or dry impregnation (DI), on HEK293 (A,C) and PC3 (B,D) cells as a function of Zn content, assessed by MTT assay after 24 h incubation. Results are presented as mean ± SD from six values obtained in at least three independent experiments.

## Abbreviations

The following abbreviations are used in this manuscript:

ATR	Attenuated Total Reflection
BET	Brunauer–Emmett–Teller (surface area analysis method)
CFU	Colony Forming Units
CLSI	Clinical and Laboratory Standards Institute
CO_2_e	Carbon Dioxide Equivalent
DI	Dry Impregnation
EDS	Energy-Dispersive X-ray Spectroscopy
FBS	Fetal Bovine Serum
FTIR	Fourier Transform Infrared Spectroscopy
HAADF-STEM	High-Angle Annular Dark Field Scanning Transmission Electron Microscopy
HCl	Hydrochloride
HEK293	Human Embryonic Kidney 293 Cells
HRTEM	High-Resolution Transmission Electron Microscopy
KIT-6	Korea Institute of Science and Technology-6
LCA	Life Cycle Assessment
LCI	Life Cycle Inventory
MBC	Minimum Bactericidal Concentration
MIC	Minimum Inhibitory Concentration
MSU-X	Michigan State University-X
MTT	3-(4,5-Dimethylthiazol-2-yl)-2,5-diphenyltetrazolium bromide (cell viability reagent)
NLDFT	Nonlocal Density Functional Theory (pore size distribution model)
NPs	Nanoparticles
PBS	Phosphate-Buffered Saline
PC3	Human Prostate Cancer Cells
PEI	Hyperbranched Polyethyleneimine
PSD	Pore Size Distribution
ROS	Reactive Oxygen Species
RT	Room Temperature
*S. aureus*	*Staphylococcus aureus*
SBA-15	Santa Barbara Amorphous-15
SBA-16	Santa Barbara Amorphous-16
SEM	Scanning Electron Microscopy
SiO_2_	Silicon Dioxide
SSA	Specific Surface Area
STEM	Scanning Transmission Electron Microscopy
TGA	Thermogravimetric Analysis
TEM	Transmission Electron Microscopy
TEOS	Tetraethyl Orthosilicate
TPV	Total Pore Volume
TSB	Tryptic soy broth
TrP	Carboxy-Methylated Hyperbranched Polyethyleneimine (Trilon-P)
WI	Wet Impregnation
UV–vis	Ultraviolet-visible spectroscopy
XRD	X-ray Diffraction
ZnO	Zinc Oxide
