# Supplementary material for: Engineering Mesoporous Silica Hosts for Ultrasmall ZnO Nanoparticles: A Dendritic Polymer-Assisted Strategy Towards Sustainable, Safe, and Effective Antibacterial Systems
Source: Nanomaterials (Basel). 2025 Nov 9;15(22):1697. doi: 10.3390/nano15221697 (PMC12655569; doi:10.3390/nano15221697)
Supplement: Supplementary file 1 [file nanomaterials-15-01697-s001.zip › nanomaterials-3947496-supplementary.pdf]

Supporting information

Engineering Mesoporous Silica Hosts for Ultrasmall ZnO Nanoparticles: A Dendritic Polymer-Assisted Strategy Towards Sustainable, Safe and Effective Antibacterial Systems

Aggeliki Papavasiliou[1,*], Kyriaki Marina Lyra[1], Elias Sakellis [1,2], Milena Nasner[3], Jose Gallego[3], Fotios K. Katsaros[1], and Zili Sideratou[1,*]

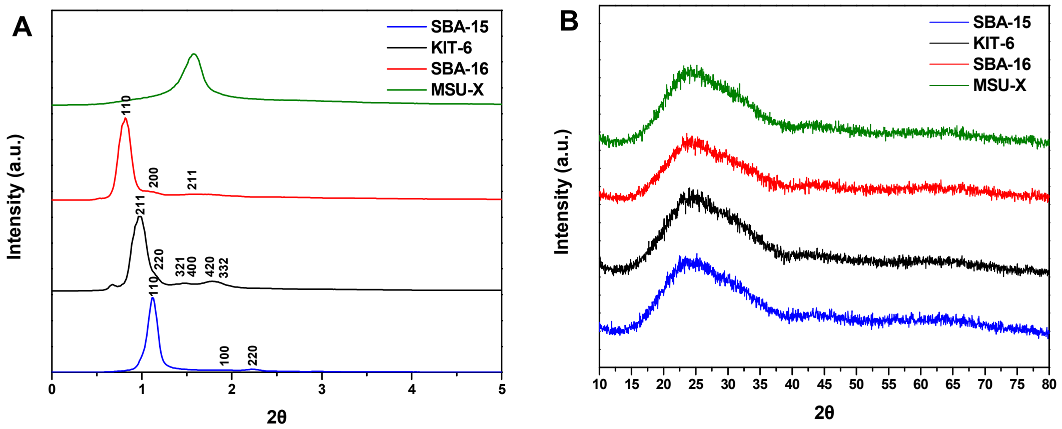

Figure S1. Wide (**A**) and Low angle XRD patterns (**B**) of pristine mesoporous silica samples.

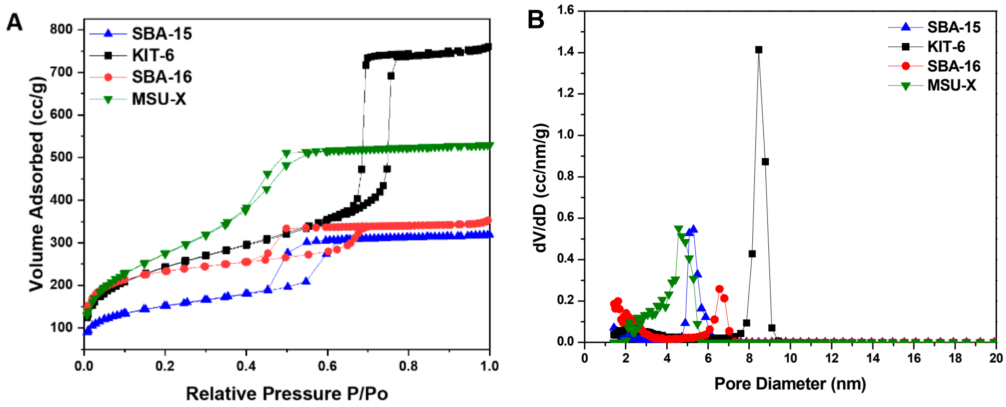

Figure S2. N_2-physisorption isotherms (**A**) and Pore Size Distribution Curves (**B**) of pure silicas.

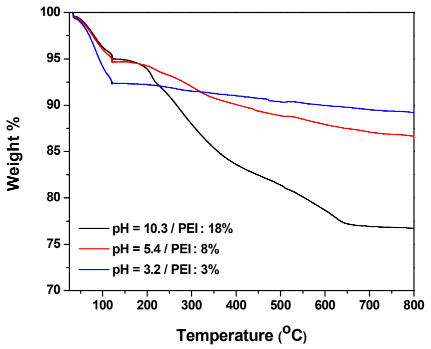

Figure S3. TGA profiles of SBA-15 sample loaded with PEI through wet impregnation at different pH. values.

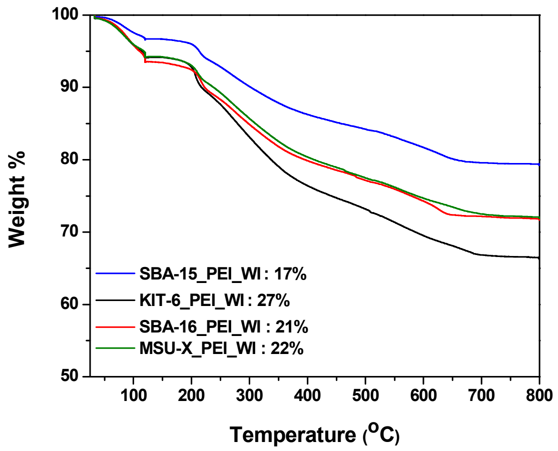

Figure S4. TGA profiles of mesoporous silicas loaded with PEI via wet impregnation.

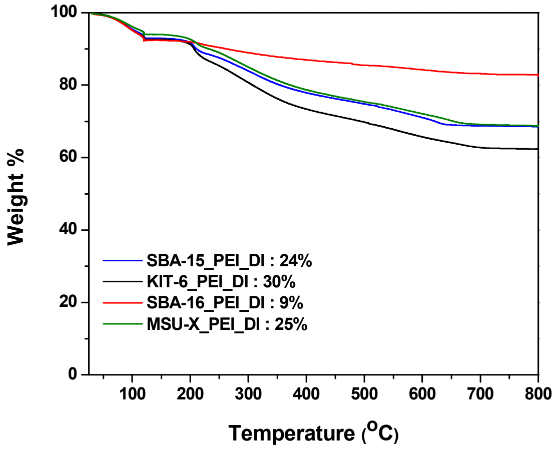

Figure S5. TGA profiles of mesoporous silicas loaded with PEI via dry impregnation.

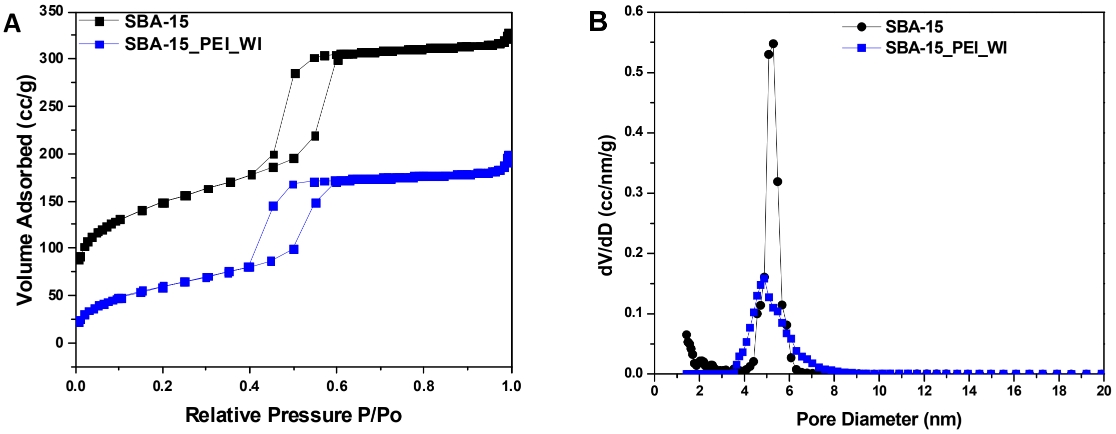

Figure S6. N_2-physisorption isotherms (**A**) and Pore Size Distribution Curves (**B**) of pure SBA-15 and PEI-loaded SBA-15 via wet impregnation (SBA15_PEI_WI).

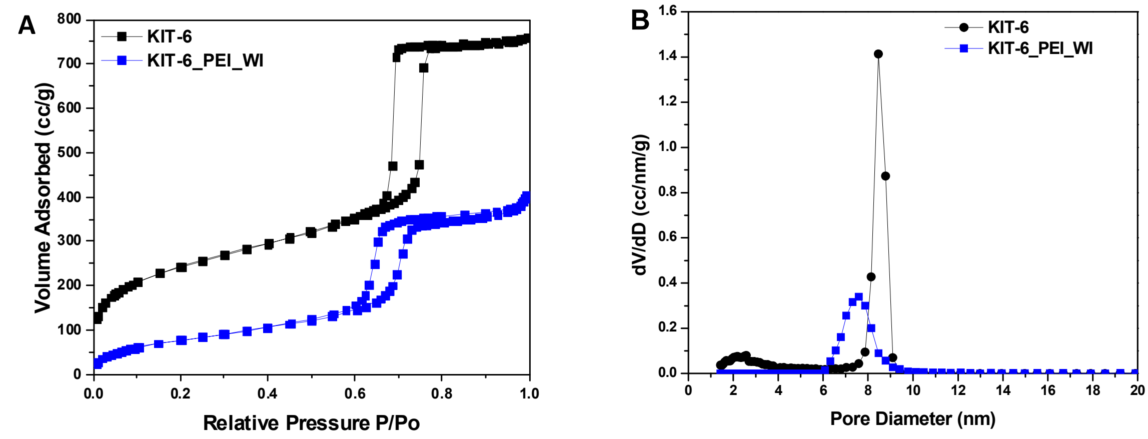

Figure S7. N_2-physisorption isotherms (**A**) and Pore Size Distribution Curves (**B**) of pure KIT-6 and PEI-loaded KIT-6 via wet impregnation (KIT-6_PEI_WI).

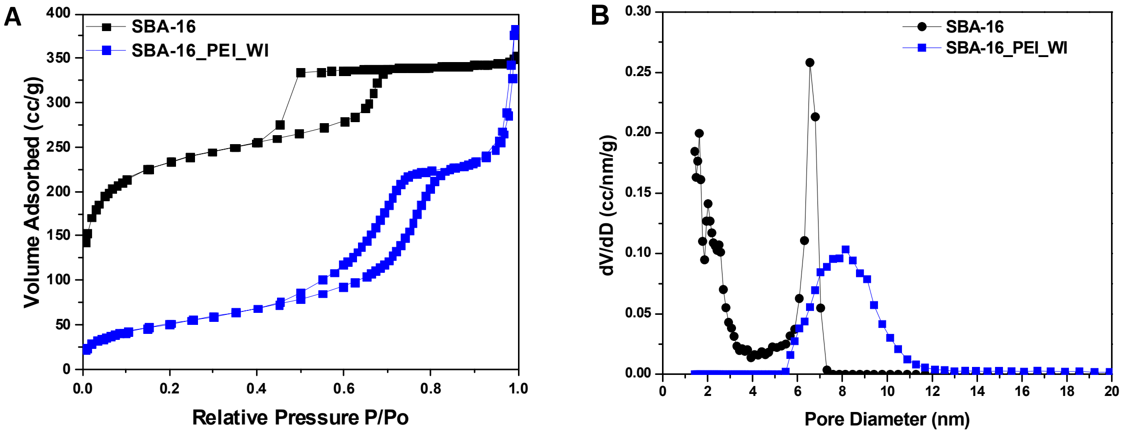

Figure S8. N_2-physisorption isotherms (**A**) and Pore Size Distribution Curves (**B**) of pure SBA-16 and PEI-loaded SBA-16 via wet impregnation (SBA-16_PEI_WI).

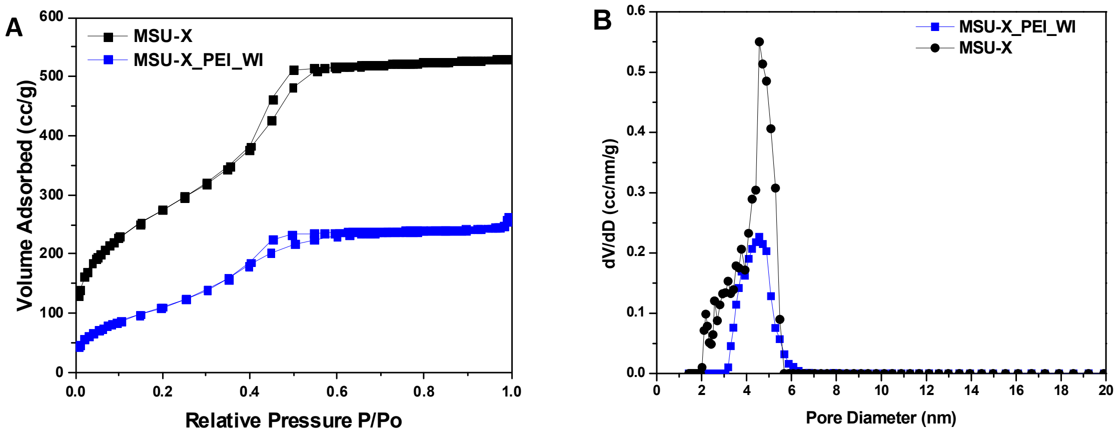

Figure S9. N_2-physisorption isotherms (**A**) and Pore Size Distribution Curves (**B**) of pure MSU-X and PEI-loaded MSU-X via wet impregnation (MSU-X_PEI_WI).

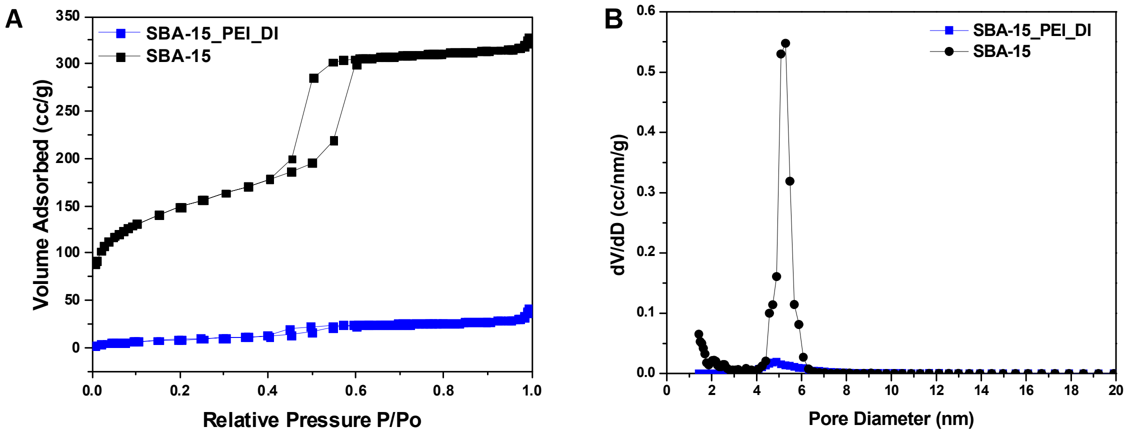

Figure S10. N_2-physisorption isotherms (A) and Pore Size Distribution Curves (B) of pure SBA-15 and PEI-loaded SBA-15 via dry impregnation (SBA-15_PEI_DI).

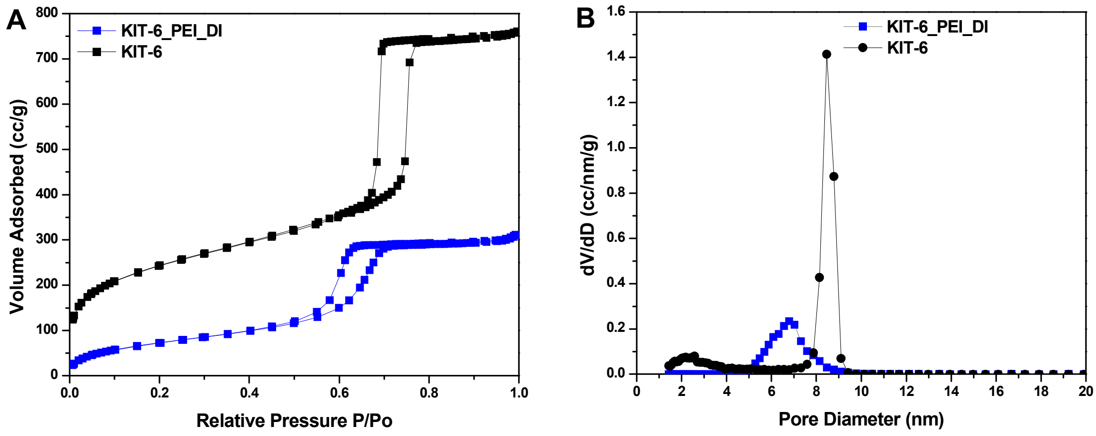

Figure S11. N_2-physisorption isotherms (**A**) and Pore Size Distribution Curves (**B**) of pure KIT-6 and PEI-loaded KIT-6 via dry impregnation (KIT-6_PEI_DI).

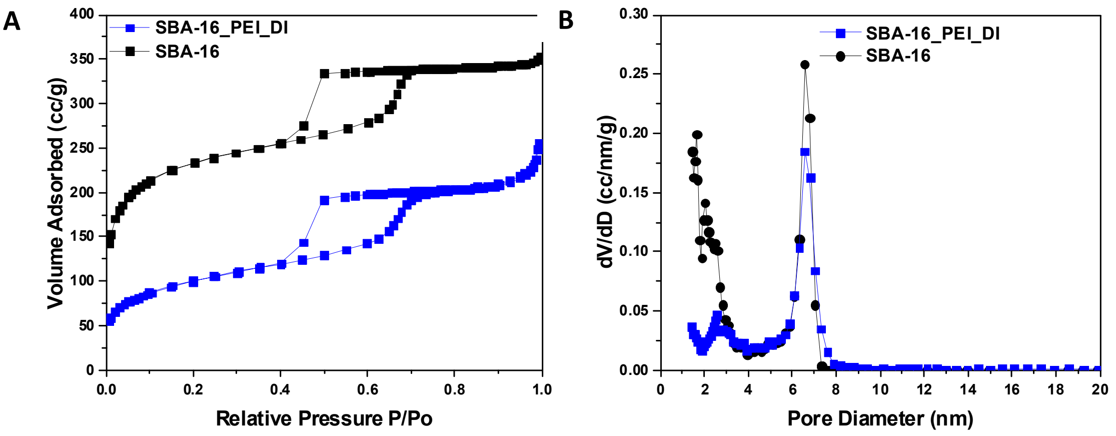

Figure S12. N_2-physisorption isotherms (**A**) and Pore Size Distribution Curves (**B**) of pure SBA-16 and PEI-loaded SBA-16 via dry impregnation (SBA-16_PEI_DI).

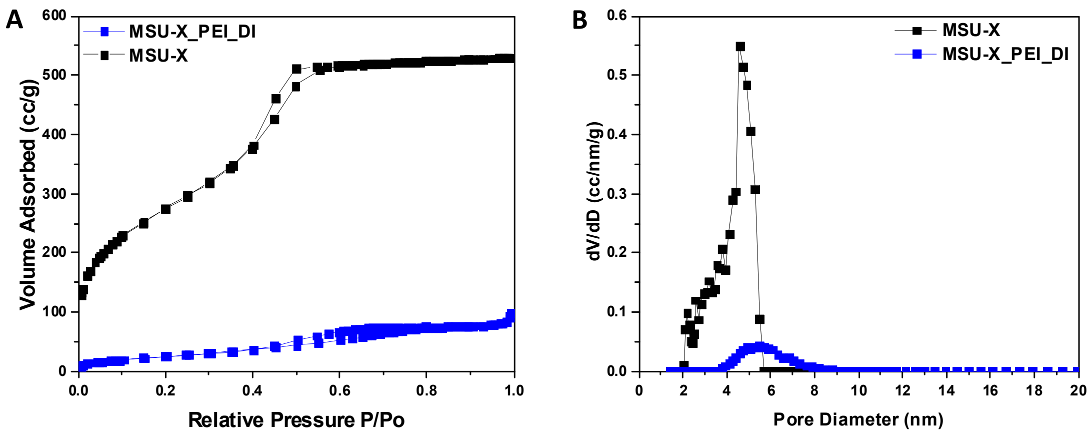

Figure S13. N_2-physisorption isotherms (**A**) and Pore Size Distribution Curves (**B**) of pure MSU-X and PEI-loaded MSU-X via dry impregnation (MSU-X_PEI_DI).

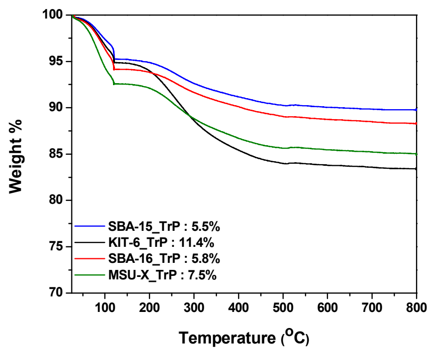

Figure S14. TGA profiles of mesoporous silicas loaded with Trilon-P (TrP) via wet impregnation.

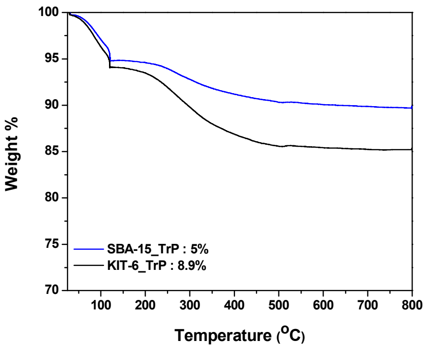

Figure S15. TGA profiles of mesoporous silicas loaded with Trilon-P via dry impregnation.

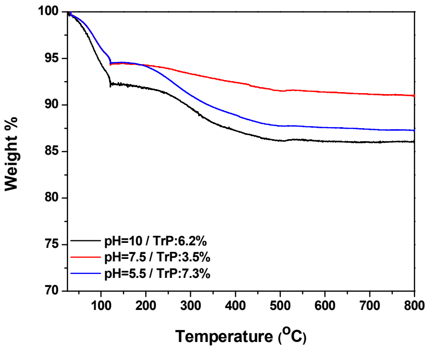

Figure S16: TGA profiles of SBA-15 sample loaded with Trilon-P (TrP) through wet impregnation at different pH values.

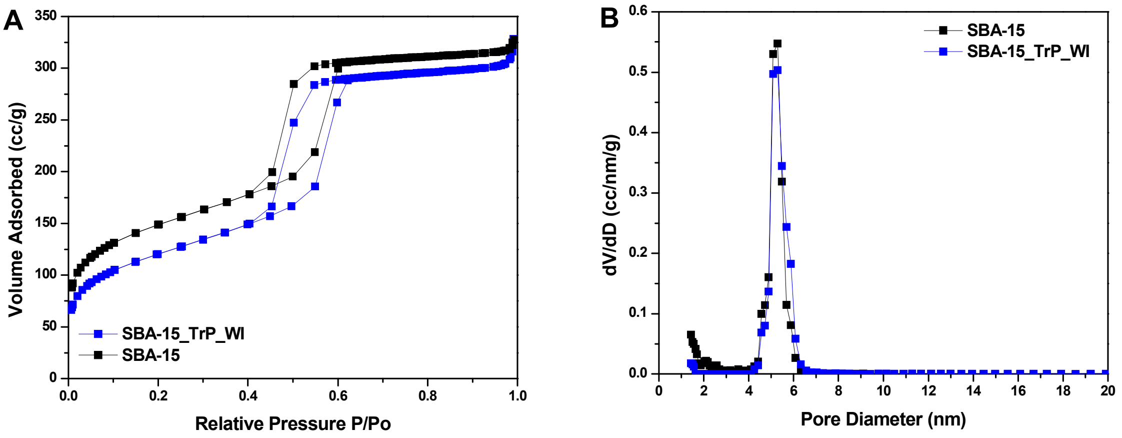

Figure S17. N_2-physisorption isotherms (**A**) and Pore Size Distribution Curves (**B**) of pure SBA-15 and Trilon-P-loaded SBA-15 via wet impregnation (SBA-15_TrP_WI).

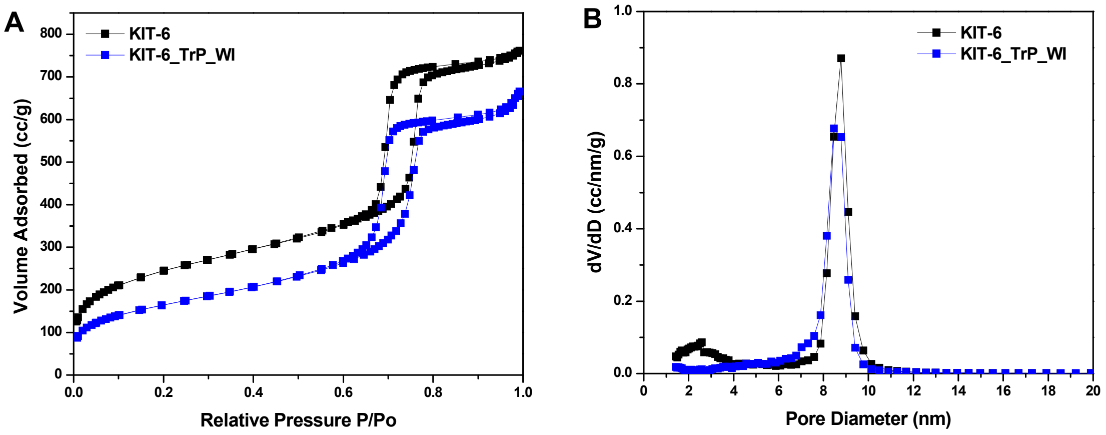

Figure S18. N2-physisorption isotherms (**A**) and Pore Size Distribution Curves (**B**) of pure KIT-6 and Trilon-P-loaded KIT-6 via wet impregnation (KIT-6_TrP_WI).

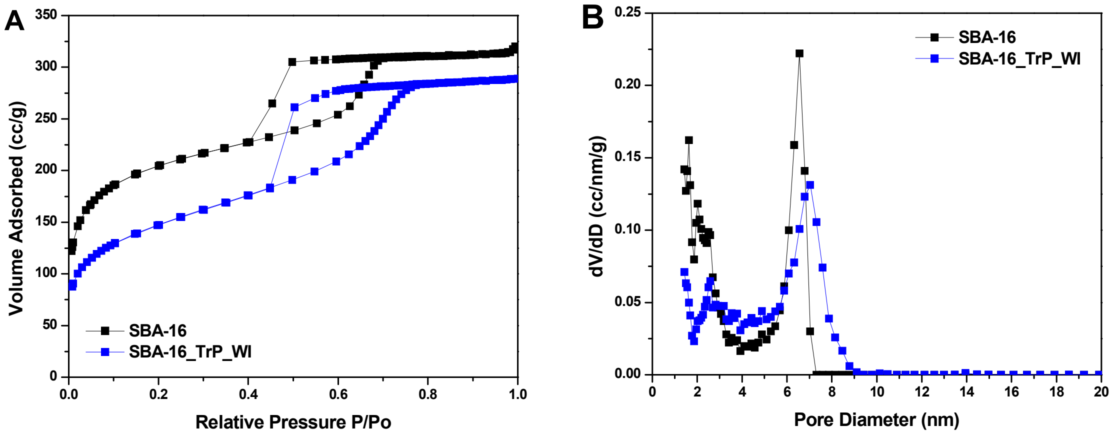

Figure S19. N2-physisorption isotherms (**A**) and Pore Size Distribution Curves (**B**) of pure SBA-16 and Trilon-P-loaded SBA-16 via wet impregnation (SBA-16_TrP_WI).

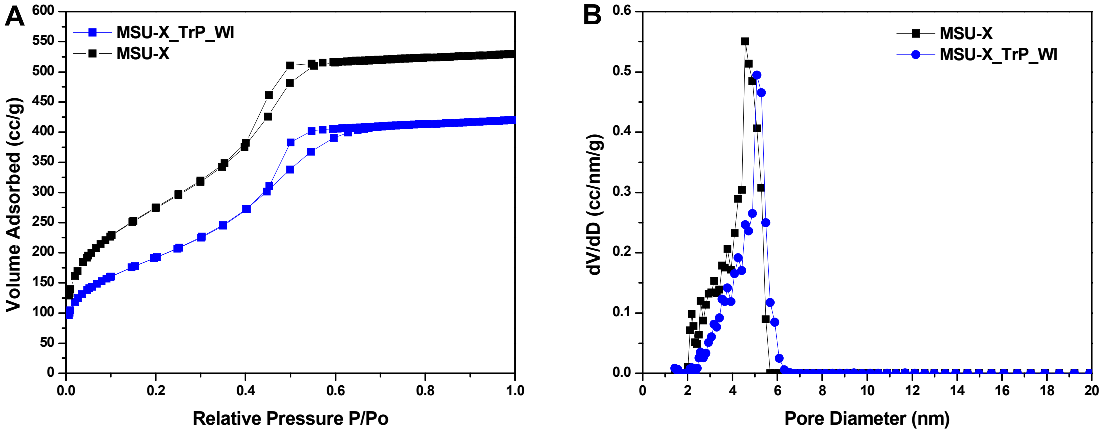

Figure S20. N2-physisorption isotherms (**A**) and Pore Size Distribution Curves (**B**) of pure MSU-X and Trilon-P-loaded MSU-X via wet impregnation (MSU-X_TrP_WI).

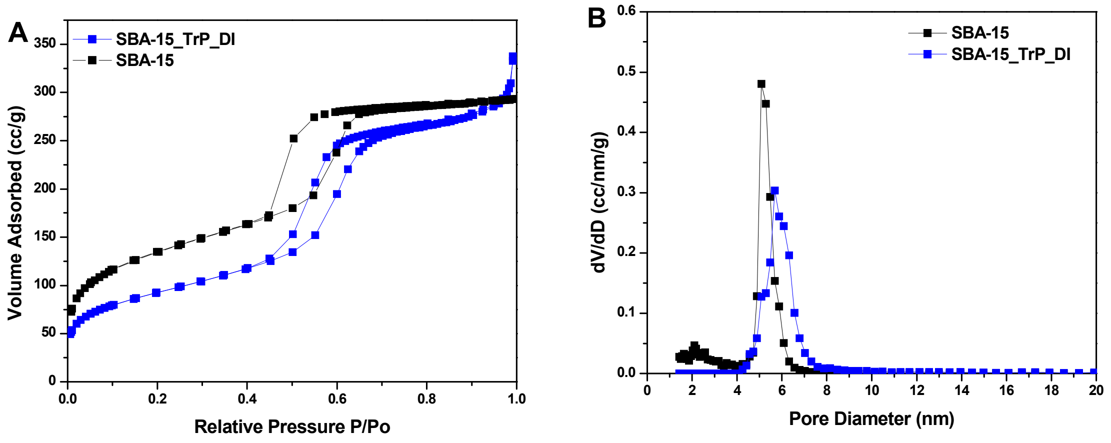

Figure S21. N_2-physisorption isotherms (**A**) and Pore Size Distribution Curves (**B**) of pure SBA-15 and Trilon-P-loaded SBA-15 via dry impregnation (SBA-15_TrP_DI).

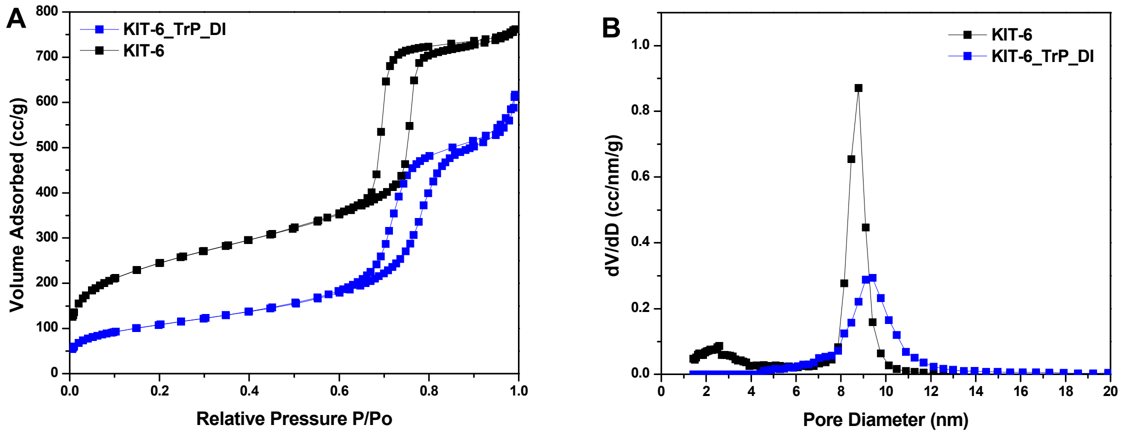

Figure S22. N_2-physisorption isotherms (**A**) and Pore Size Distribution Curves (**B**) of pure KIT-6 and Trilon-P loaded KIT-6 via dry impregnation (KIT-6_TrP_DI).

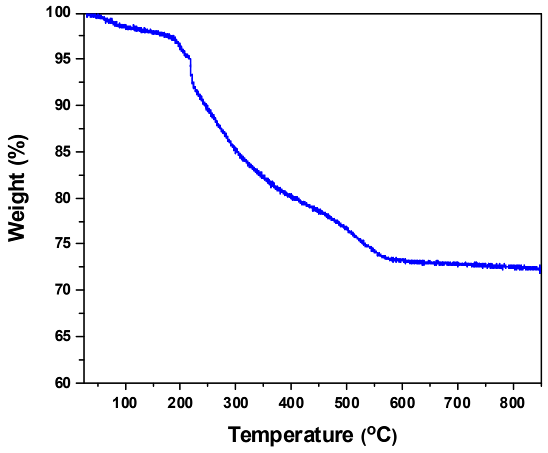

Figure S23. TGA profile of the dried ZnO/SBA-15_PEI_DI.

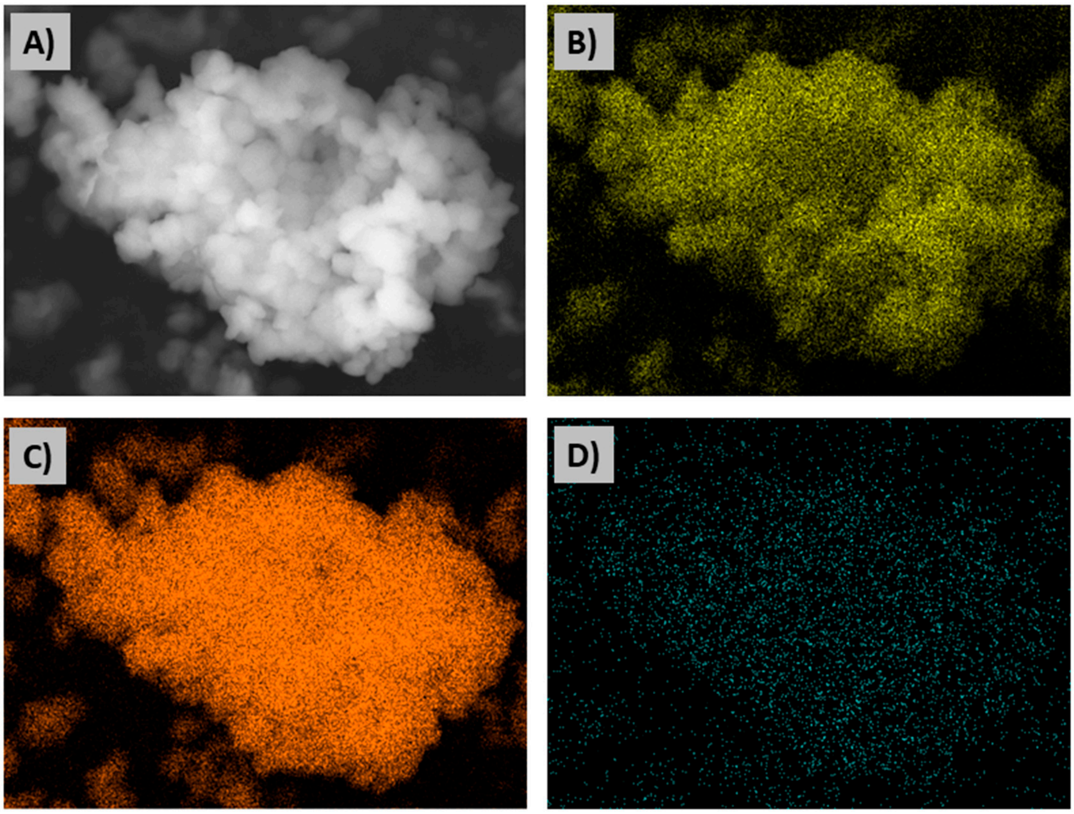

Figure S24. (**A**) SEM micrograph of ZnO/SBA-15_PEI_DI and the corresponding (**B**) O, (**C**) Si and (**D**) Zn elemental maps.

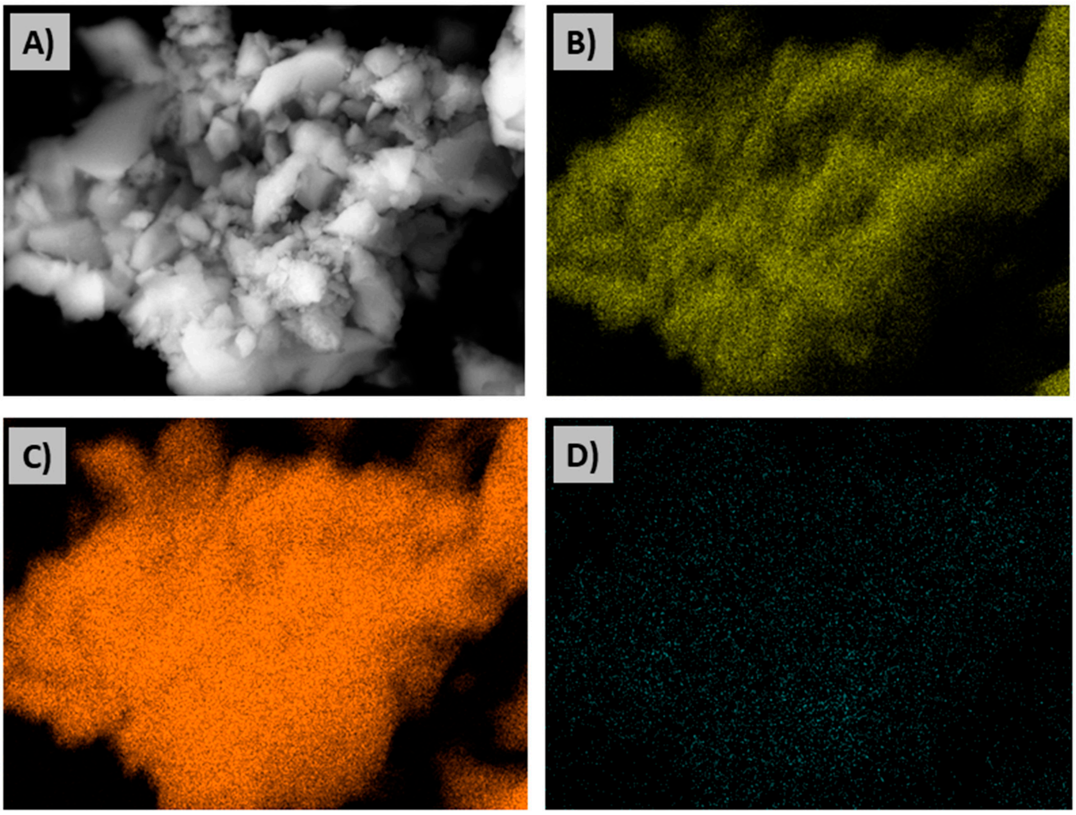

Figure S25. (**A**) SEM micrograph of ZnO/SBA-16_PEI_DI and the corresponding (**B**) O, (**C**) Si and (**D**) Zn elemental maps.

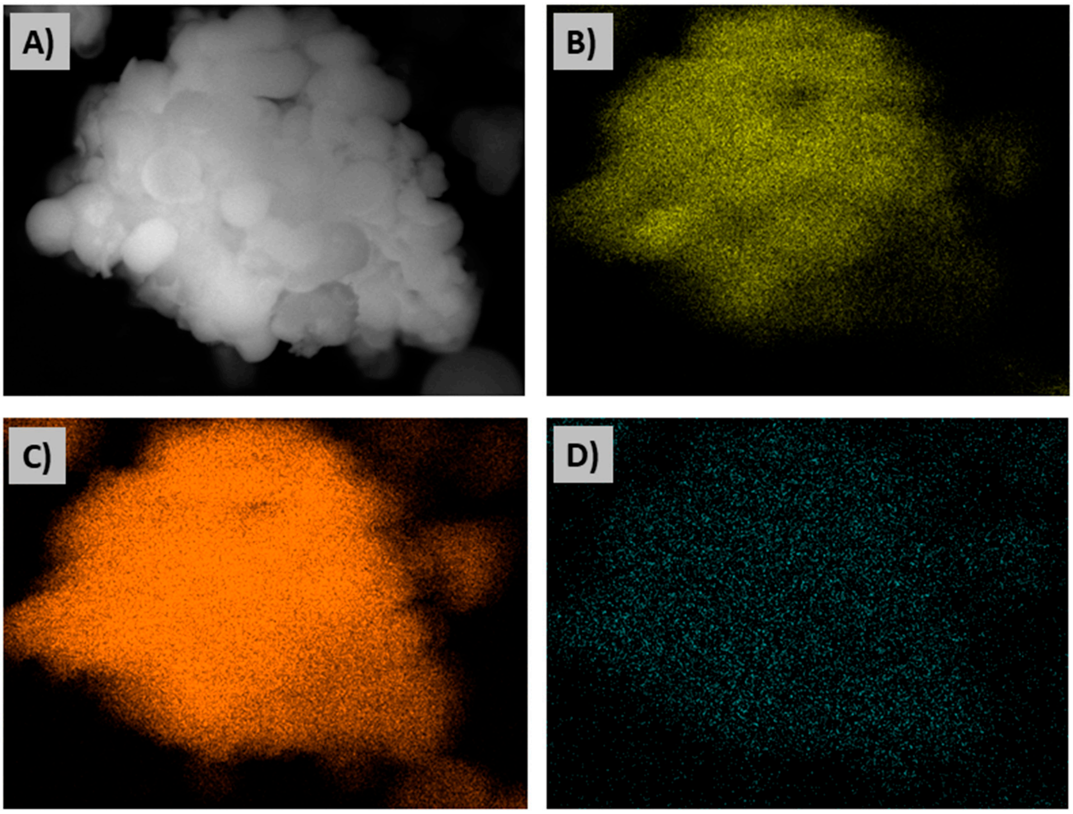

Figure S26. (**A**) SEM micrograph of ZnO/MSU-X_PEI_DI and the corresponding (**B**) O, (**C**) Si and (**D**) Zn elemental maps.

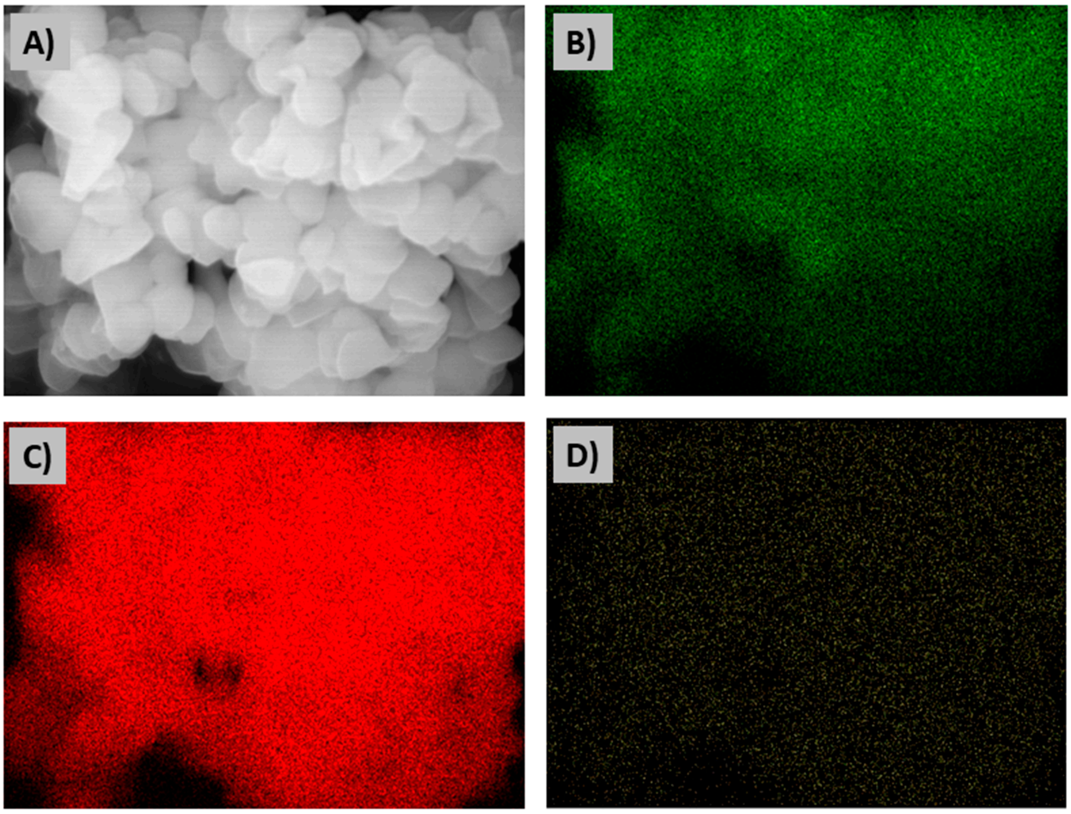

Figure S27. (**A**) SEM micrograph of ZnO/SBA-15_PEI_WI sample and the corresponding (**B**) O, (**C**) Si and (**D**) Zn elemental maps.

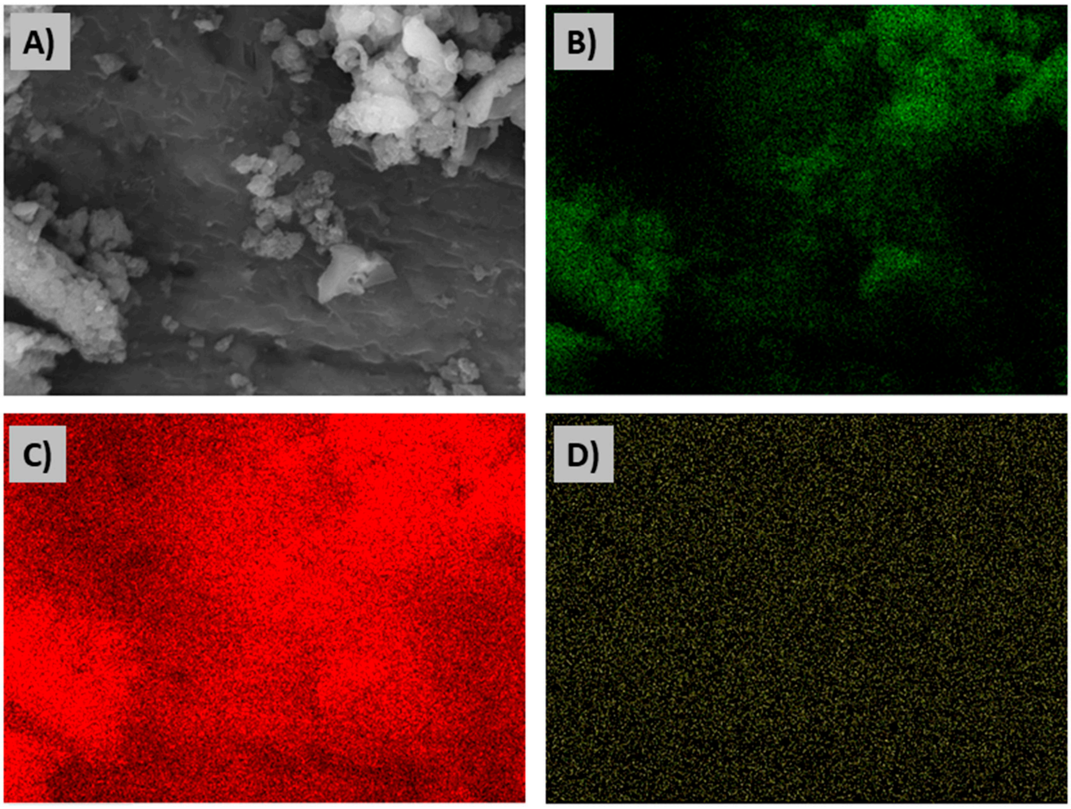

Figure S28. (**A**) SEM micrograph of ZnO/KIT-6_PEI_WI sample and the corresponding (**B**) O, (**C**) Si and (**D**) Zn elemental maps.

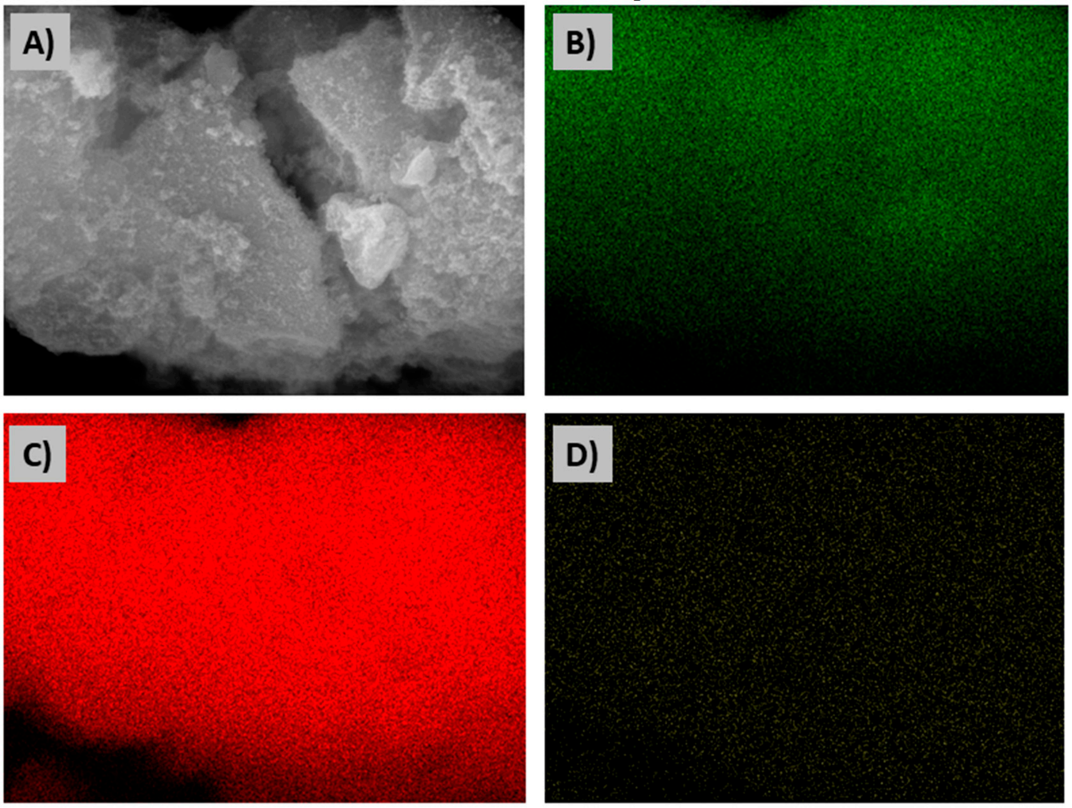

Figure S29. (**A**) SEM micrograph of ZnO/SBA-16_PEI_WI sample and the corresponding (**B**) O, (**C**) Si and (**D**) Zn elemental maps.

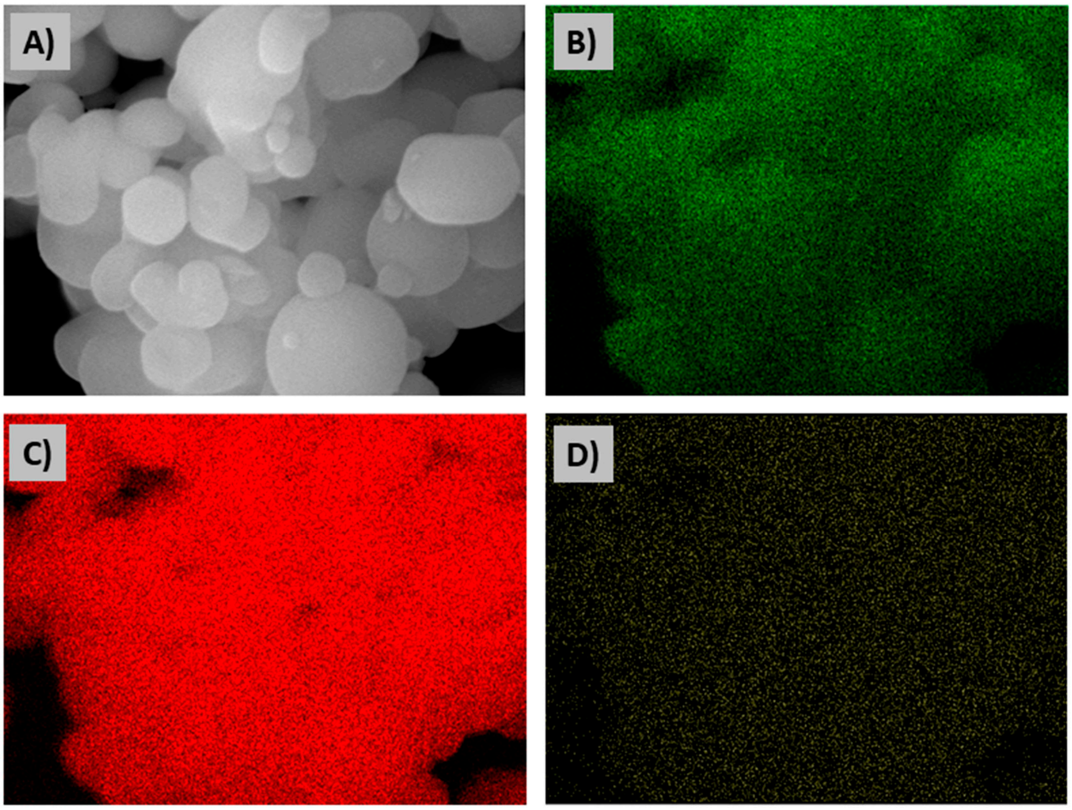

Figure S30. (**A**) SEM micrograph of ZnO/MSU-X_PEI_WI sample and the corresponding (B) O, (**C**) Si and (**D**) Zn elemental maps.

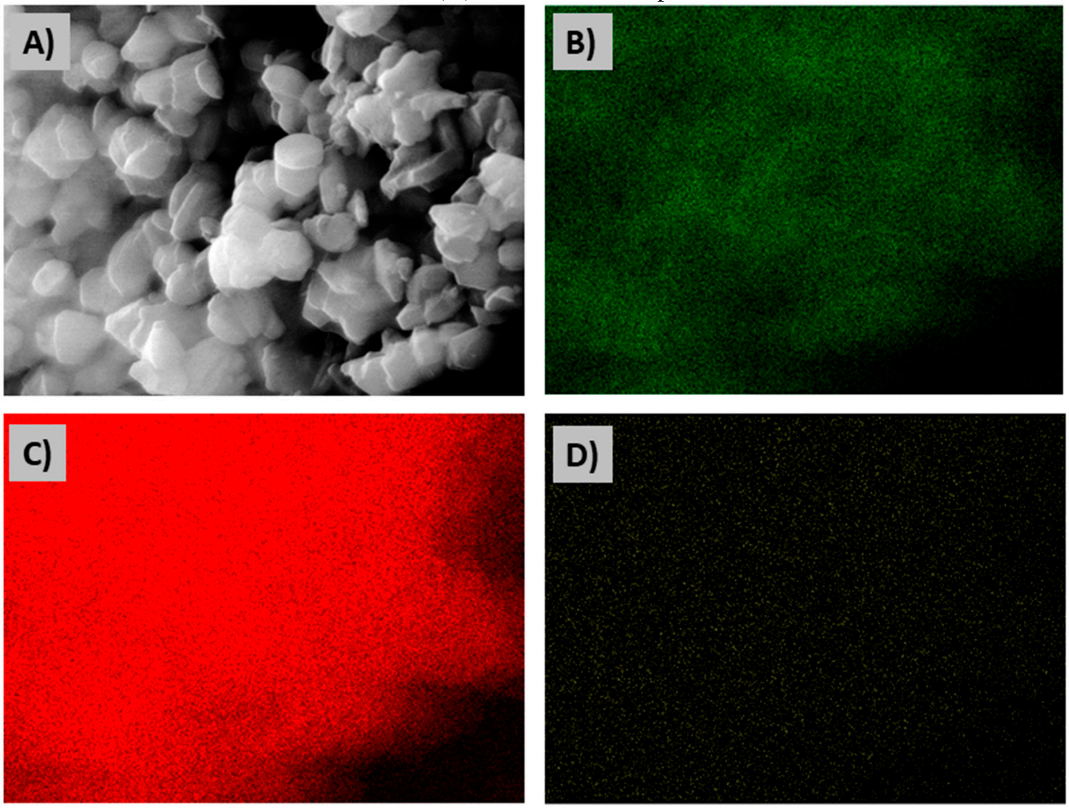

Figure S31. (**A**) SEM micrograph of ZnO/SBA-15_TrP_WI sample and the corresponding (**B**) O, (**C**) Si and (**D**) Zn elemental maps.

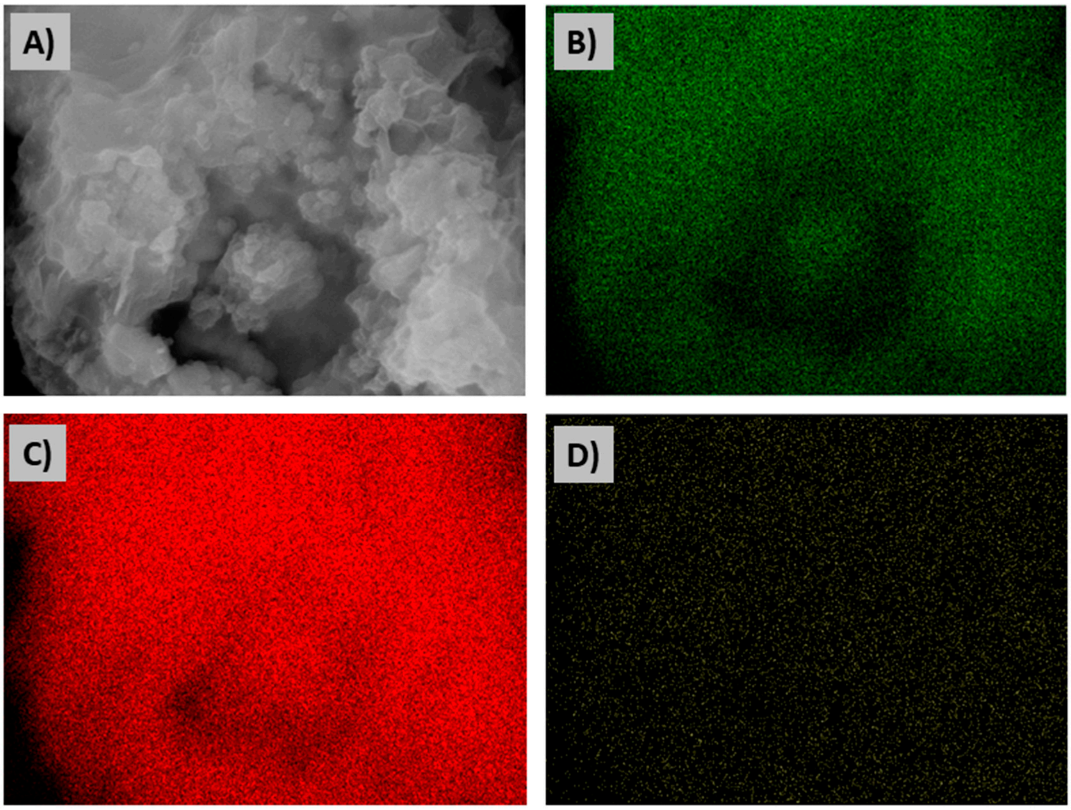

Figure S32. (**A**) SEM micrograph of ZnO/KIT-6_TrP_WI sample and the corresponding (**B**) O, (**C**) Si and (**D**) Zn elemental maps.

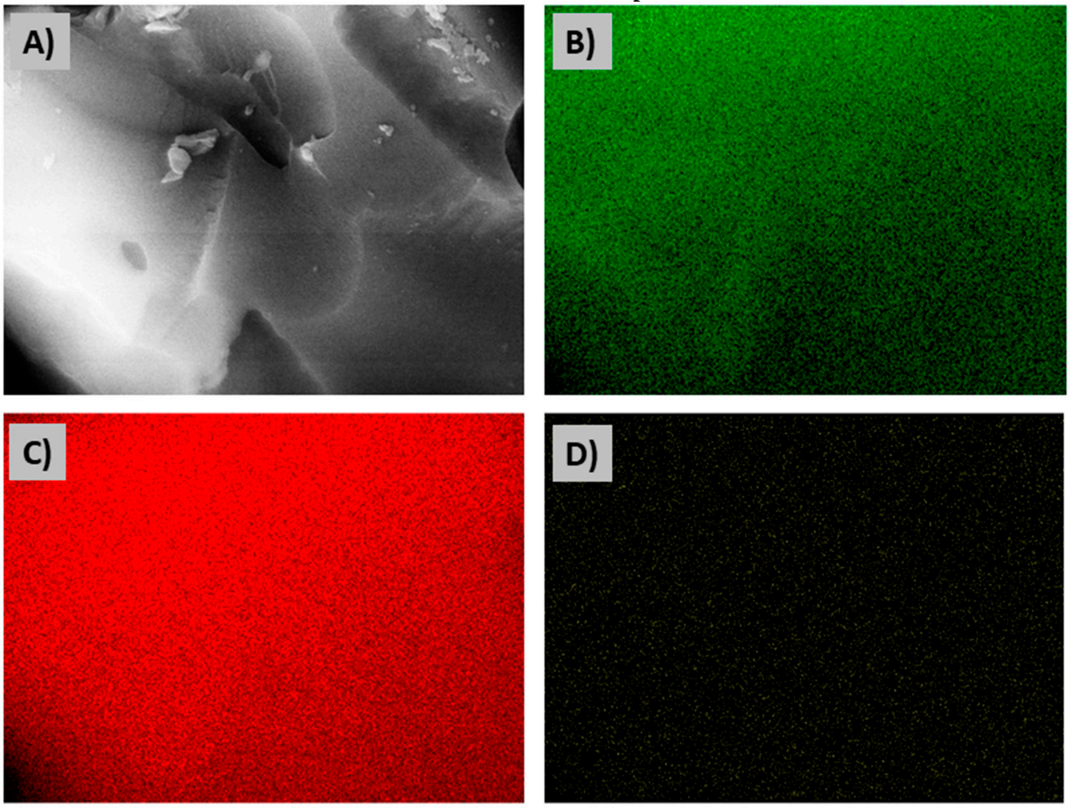

Figure S33. (**A**) SEM micrograph of ZnO/SBA-16_TrP_WI sample and the corresponding (**B**) O, (**C**) Si and (**D**) Zn elemental maps.

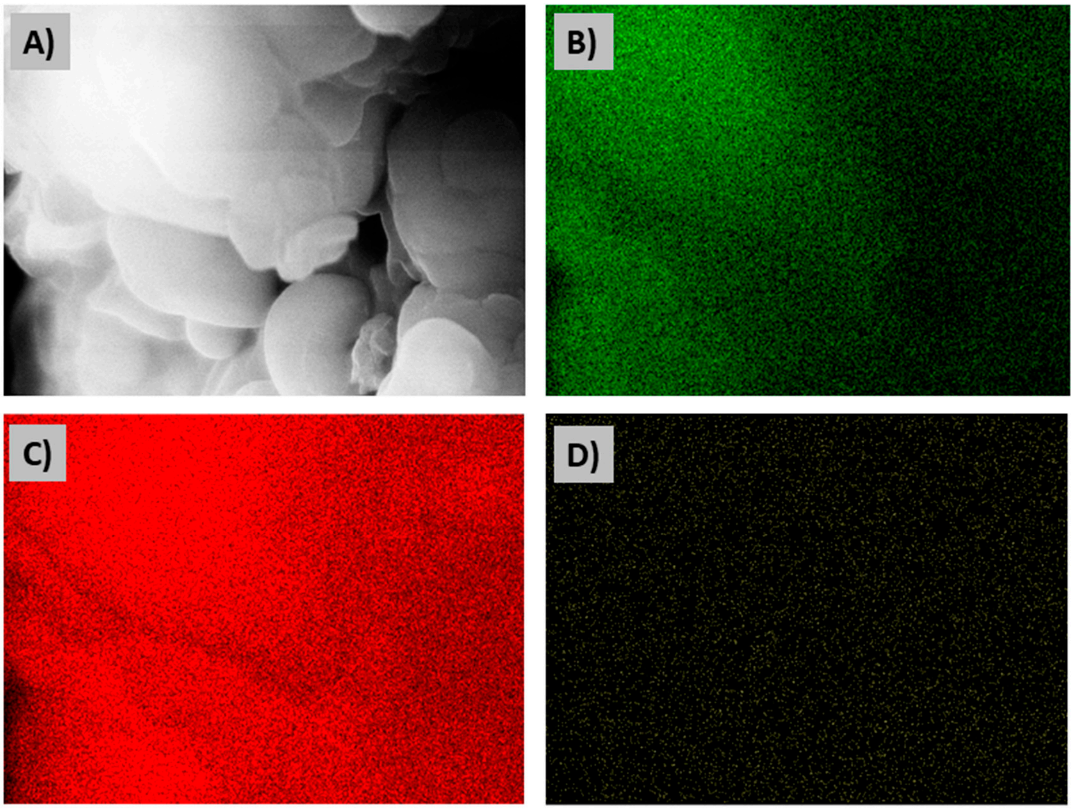

Figure S34. (**A**) SEM micrograph of ZnO/MSU-X_Trilon-P_WI sample and the corresponding (**B**) O, (**C**) Si and (**D**) Zn elemental maps.

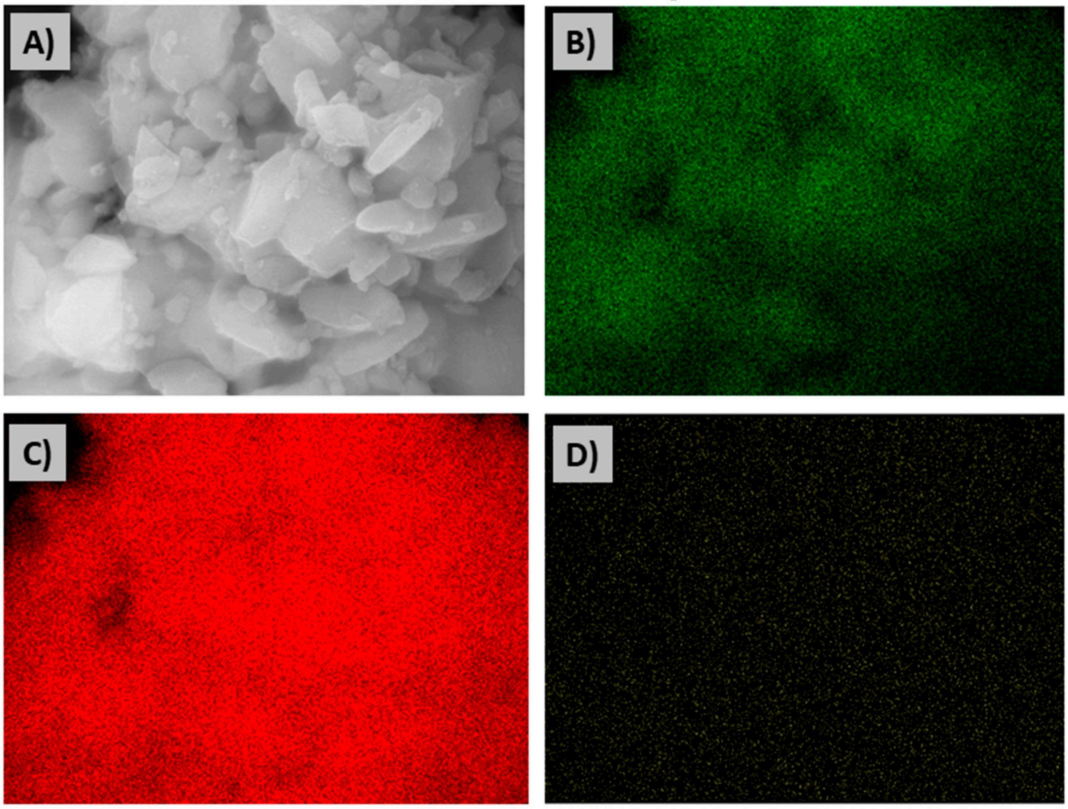

Figure S35. (**A**) SEM micrograph of ZnO/SBA-15_TrP_DI sample and the corresponding (**B**) O, (**C**) Si and (**D**) Zn elemental maps.

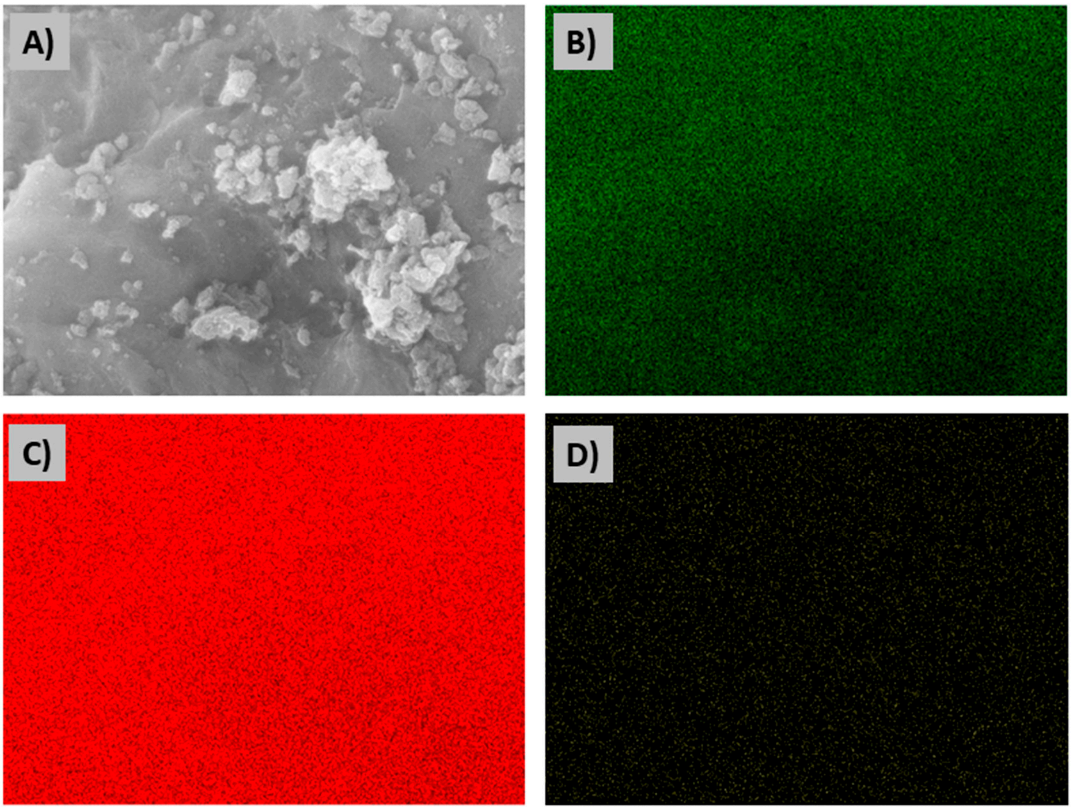

Figure S36. (**A**) SEM micrograph of ZnO/KIT-6_TrP_DI sample and the corresponding (**B**) O, (**C**) Si and (**D**) Zn elemental maps.

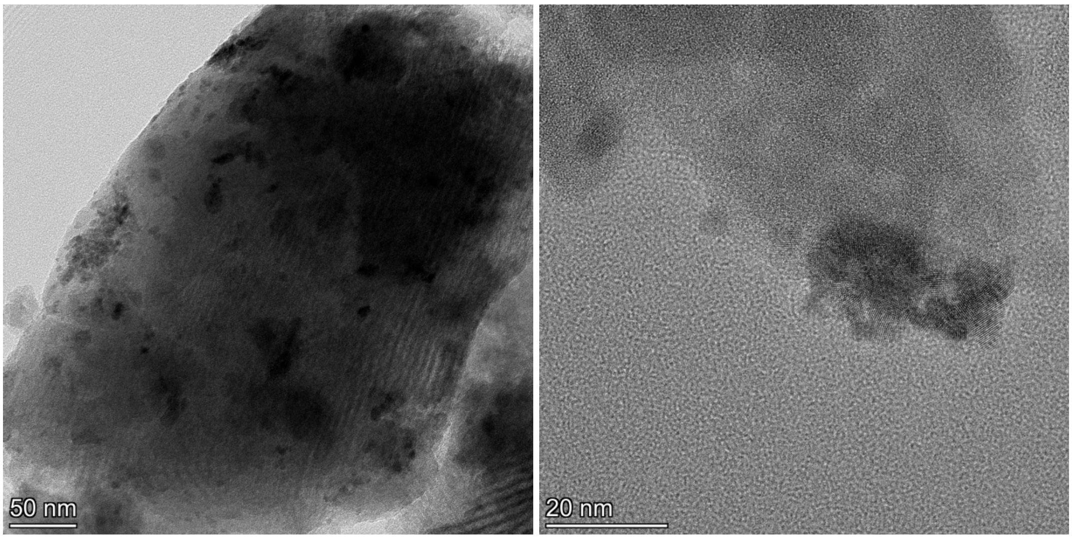

Figure S37. TEM micrographs of ZnO/SBA-15_PEI_DI sample.

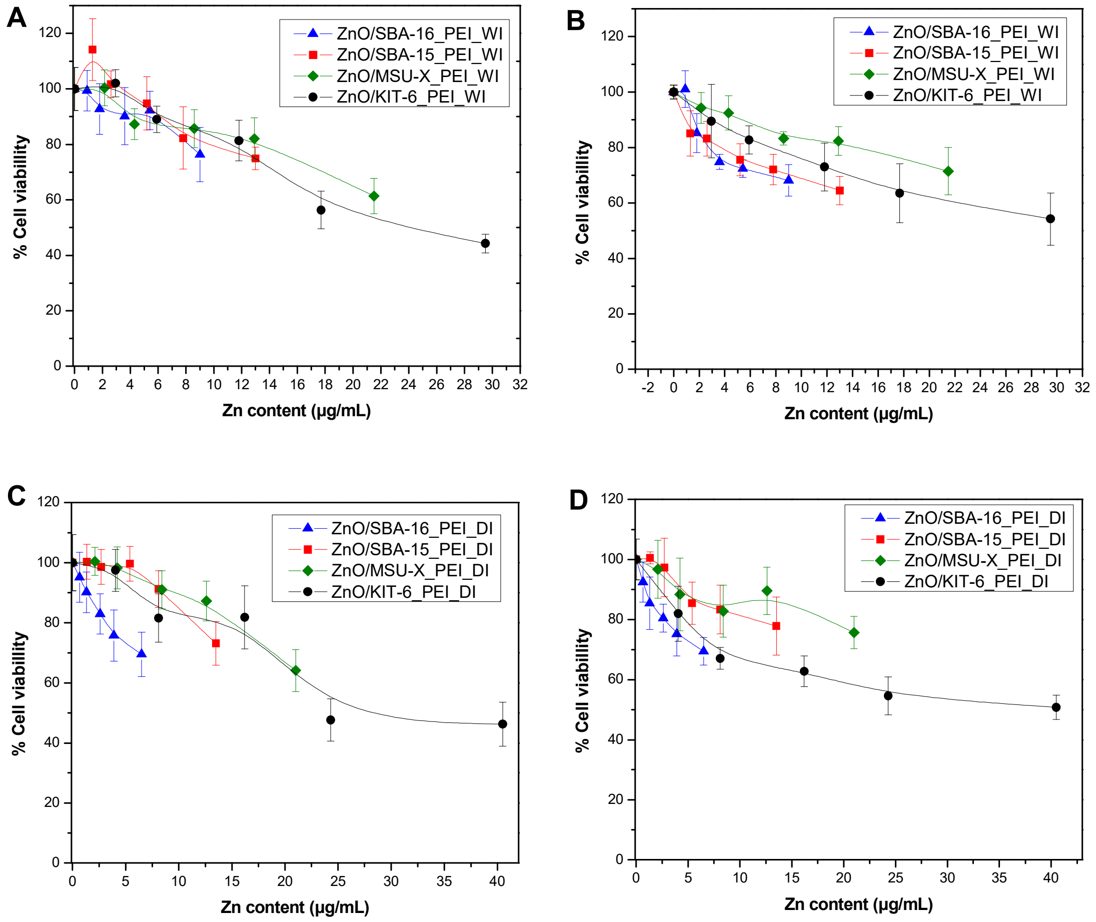

Figure S38. Comparative cytotoxicity of ZnO-loaded mesoporous silica nanocomposites via PEI, using either wet impregnation (WI) or dry impregnation (DI), on HEK293 (**A**, **C**) and PC3 (**B**, **D**) cells as a function of Zn content, assessed by MTT assay after 24 h incubation. Results are presented as mean ± SD from six values obtained in at least three independent experiments.

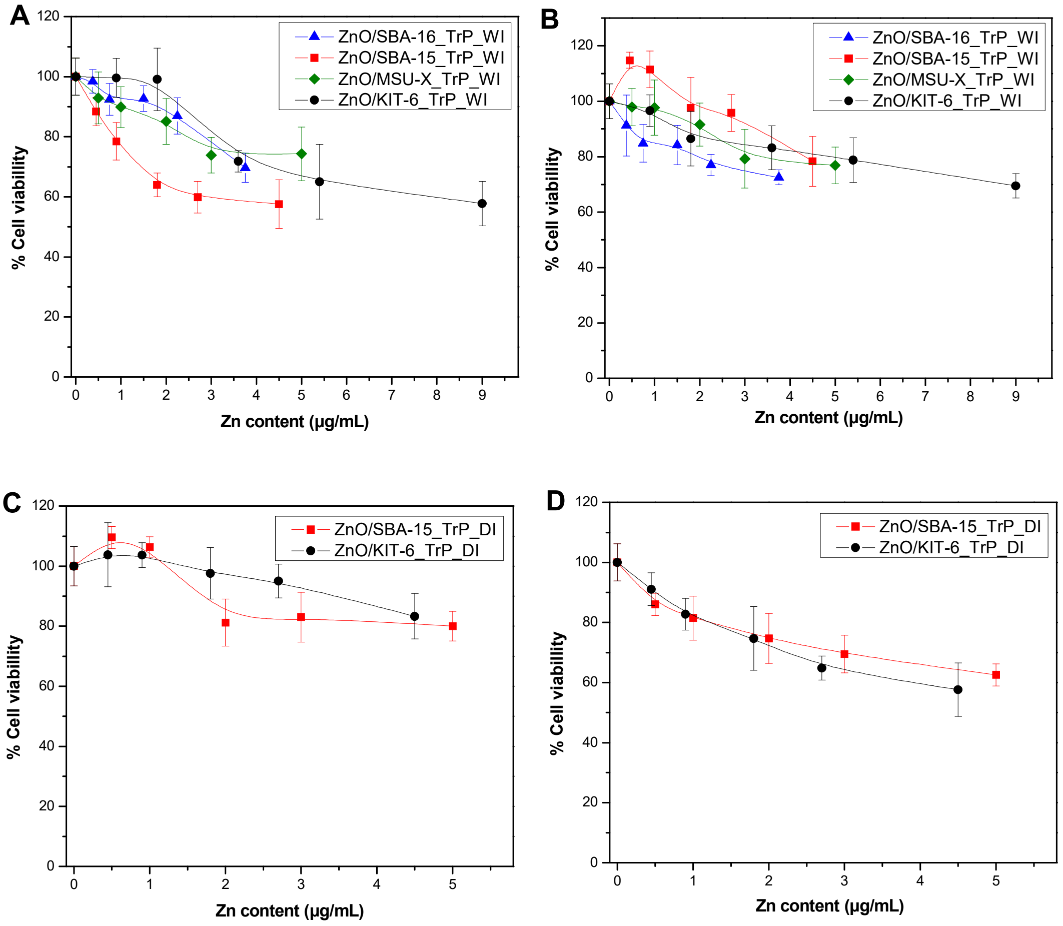

Figure S39. Comparative cytotoxicity of ZnO-loaded mesoporous silica nanocomposites via TrP, using either wet impregnation (WI) or dry impregnation (DI), on HEK293 (**A**, **C**) and PC3 (**B**, **D**) cells as a function of Zn content, assessed by MTT assay after 24 h incubation. Results are presented as mean ± SD from six values obtained in at least three independent experiments.
